# β-glucan–dependent shuttling of conidia from neutrophils to macrophages occurs during fungal infection establishment

**DOI:** 10.1371/journal.pbio.3000113

**Published:** 2019-09-04

**Authors:** Vahid Pazhakh, Felix Ellett, Ben A. Croker, Joanne A. O’Donnell, Luke Pase, Keith E. Schulze, R. Stefan Greulich, Aakash Gupta, Constantino Carlos Reyes-Aldasoro, Alex Andrianopoulos, Graham J. Lieschke

**Affiliations:** 1 Australian Regenerative Medicine Institute, Monash University, Clayton, Victoria, Australia; 2 Cancer and Haematology Division, Walter and Eliza Hall Institute of Medical Research, Parkville, Victoria, Australia; 3 Department of Medical Biology, University of Melbourne, Parkville, Victoria, Australia; 4 Boston Children’s Hospital, Harvard Medical School, Boston, Massachusetts, United States of America; 5 Department of Pediatrics, University of California San Diego, La Jolla, California, United States of America; 6 Monash Micro Imaging, Monash University, Clayton, Victoria, Australia; 7 Genetics, Genomics and Systems Biology, School of BioSciences, University of Melbourne, Parkville, Victoria, Australia; 8 School of Mathematics, Computer Science and Engineering, City University of London, London, United Kingdom; Carnegie Mellon University, UNITED STATES

## Abstract

The initial host response to fungal pathogen invasion is critical to infection establishment and outcome. However, the diversity of leukocyte–pathogen interactions is only recently being appreciated. We describe a new form of interleukocyte conidial exchange called “shuttling.” In *Talaromyces marneffei* and *Aspergillus fumigatus* zebrafish in vivo infections, live imaging demonstrated conidia initially phagocytosed by neutrophils were transferred to macrophages. Shuttling is unidirectional, not a chance event, and involves alterations of phagocyte mobility, intercellular tethering, and phagosome transfer. Shuttling kinetics were fungal-species–specific, implicating a fungal determinant. β-glucan serves as a fungal-derived signal sufficient for shuttling. Murine phagocytes also shuttled in vitro. The impact of shuttling for microbiological outcomes of in vivo infections is difficult to specifically assess experimentally, but for these two pathogens, shuttling augments initial conidial redistribution away from fungicidal neutrophils into the favorable macrophage intracellular niche. Shuttling is a frequent host–pathogen interaction contributing to fungal infection establishment patterns.

## Abstract 2

Imaging of the behaviour of white blood cells in living zebrafish embryos infected with fungi reveals “shuttling,” a specific and previously undescribed form of microorganism exchange between neutrophils and macrophages.

## Introduction

In vertebrates, two phagocytic cell types have long been recognized as key players in the initial host defense response to infection: neutrophil granulocytes and macrophages [1]. Neutrophils and macrophages share many features: they are both migratory cells, they phagocytose microorganisms on encountering them, and they have intracellular mechanisms for killing microorganisms. However, although both phagocyte types engulf microorganisms, individual microorganisms interact with neutrophils and macrophages with different species-specific preferences, in different ways, and using different molecular mechanisms [2]. Conversely, the host has evolved diverse cellular strategies for these two different phagocytes to protect against the panoply of potentially pathogenic microorganisms.

The exchange of cytoplasmic material through contact-dependent mechanisms between adjacent cells is currently a topical field in cell biology. An example is the contact-dependent exchange of cytoplasm from macrophage to tumor cells as a metastasis-promoting mechanism [3], distinct from the cytoplasmic exchange between macrophages and tumor cells that occur via extracellular vesicles and nanotubes [4–6].

During infections, neutrophils and macrophages also engage in intercellular exchanges. Some microorganisms have evolved mechanisms that exploit these to enhance their pathogenicity and promote their spread between phagocytes. For example, *Yersinia pestis* and *Leishmania* promastigotes induce apoptosis in host neutrophils to then exploit efferocytosis, whereby clearance of dead neutrophils by macrophages leads to subsequent infection of this less-hostile host cell [7–9]. Conversely, neutrophil phagocytosis of debris from dying macrophages is a recently demonstrated method of mycobacterial dissemination [10]. *Candida albicans* [11] and *Cryptococcus neoformans* [12] can be ejected from host macrophages by nonlytic exocytosis, while macrophage-resident *C*. *neoformans* [13] and *A*. *fumigatus* [14] also can enter new host macrophages through lateral transfer (recently termed metaforosis [15]). The gram-negative bacteria *Francisella tularensi* and *Salmonella enterica* are transferred between macrophages by a process related to trogocytosis [16]. These scenarios are characterized by death of the donor cell, expulsion of the pathogen from the donor cell without direct contact between donor and recipient phagocyte, or transfer between the same type of phagocyte. None involve transfer by direct contact from a living neutrophil to a living macrophage.

Such interactions provide an opportunity for intracellular pathogens to transfer to a new host cell while minimizing exposure to a potentially hostile extracellular environment. As antibiotic resistance becomes a growing problem, there is an ever-increasing interest in host-pathway–directed anti-infective therapies. Host-dependent processes for pathogen dissemination represent key potential targets [17].

Zebrafish have emerged as an ideal model for intravital imaging of leukocyte behaviors during infection [18]. They combine the advantages of small size, optical transparency (particularly as embryos and larvae), and suitability for genetic manipulation. Zebrafish phagocytes have been comprehensively characterized in developmental, genetic, and functional studies [19].

Our recent modeling of fungal infections in zebrafish models have focused on high spatiotemporal resolution intravital imaging of the initial leukocyte–pathogen interactions [20]. During these studies, we observed a form of microorganism exchange between neutrophils and macrophages that we believe to be previously undescribed, which we have named “shuttling.” In shuttling, a living donor neutrophil laden with previously phagocytosed fungal spore(s) transfers this cargo to a recipient macrophage through a tethered direct contact, without death of the donor neutrophil. Shuttling is therefore different from all the previously described microorganism exchanges between phagocytes.

In the present study, we comprehensively describe neutrophil-to-macrophage “shuttling.” Studying shuttles presented considerable technical challenges because they could only be identified by directly observing them retrospectively in in vivo live-imaging datasets. To recognize a shuttle, all three phases of the process had to be captured in the imaged volume: initial carriage of a phagocytosed spore within a mobile, living donor neutrophil; the moment of intercellular contact and transfer between neutrophil and macrophage; and the departure of the previously unladen recipient macrophage, now carrying its newly acquired cargo. All three shuttle-defining steps needed to have occurred within the imaged volume, despite the high mobility of the participating cells. Despite this challenge, we comprehensively describe the morphology of shuttling, quantify key parameters of the dynamic transfer process, and identify a key mechanistic determinant by demonstrating that the conidial cell-wall component β-glucan is a fungal-derived molecular signal sufficient to trigger shuttling of particles. Additionally, by replicating this phenomenon using murine phagocytes in vitro, we provide evidence that shuttling is a conserved behavior of both fish and mammalian phagocytes.

## Results

### Some *T*. *marneffei* conidia phagocytosed by neutrophils are “shuttled” to macrophages

**Fig 1:**
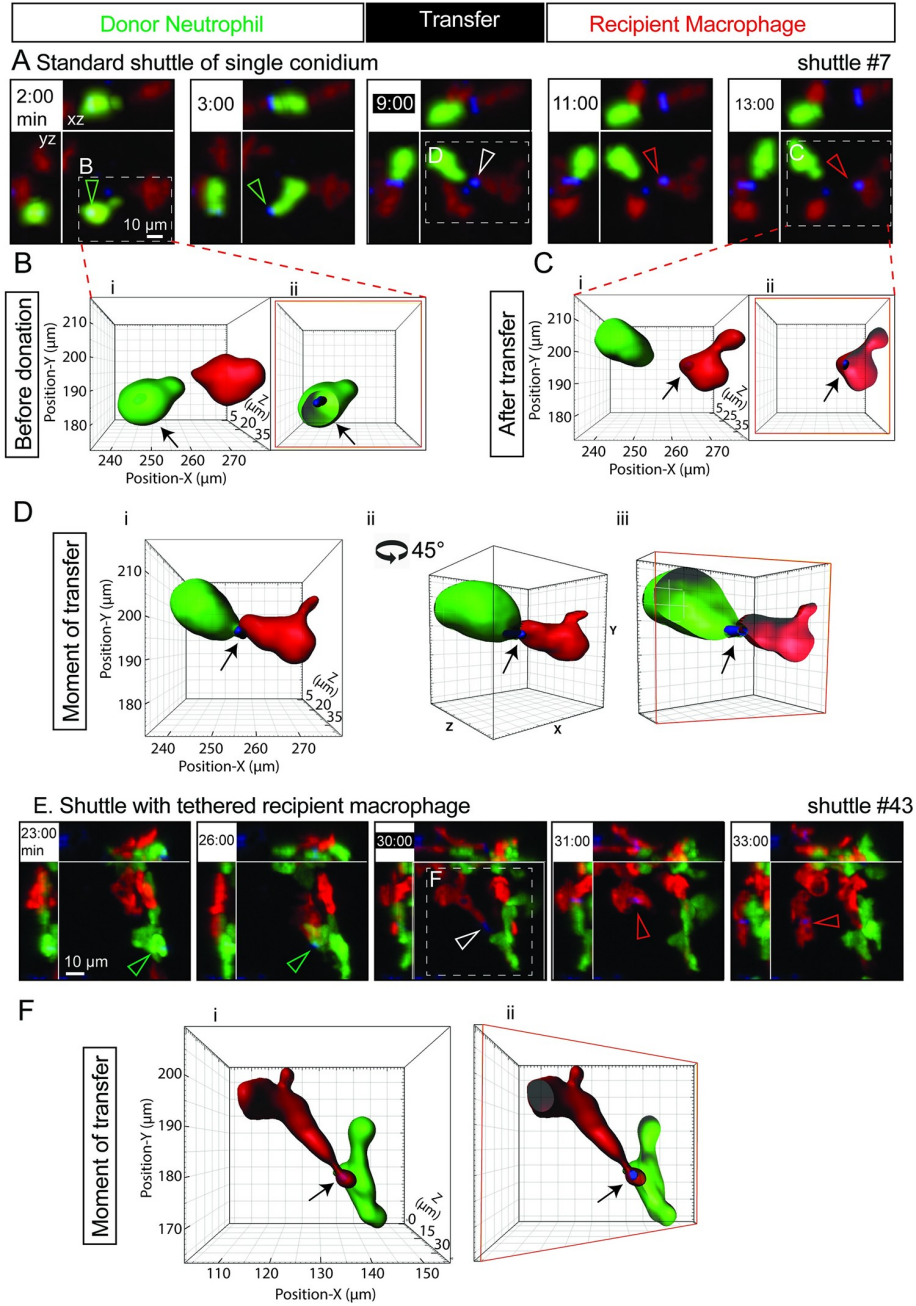
Shuttling of individual *T*. *marneffei* conidia from neutrophil to macrophage. (A) A representative standard shuttle of a calcofluor-stained conidium (blue) from a *Tg(mpx*:*EGFP)* neutrophil (green) to a *Tg(mpeg1*:*Gal4FF)×(UAS-E1b*:*Eco*.*NfsB-mCherry)* macrophage (red), corresponding to the example in S1A Movie. Panels include isometric orthogonal *yz* and *xz* views corresponding to the *xy* maximal intensity projection and indicate the time in min from start of movie. The time point colored white-on-black is the moment of transfer. Colored arrowheads indicate the conidium within donor neutrophil (green), at the point of intercellular transfer (white) and in the recipient macrophage (red). (B–D) Volume-rendered views of the standard shuttle in (A), detailed before (B), at the moment of transfer (C), and afterwards (D), demonstrating the intracellular location of the shuttled spore in donor neutrophil and recipient macrophage, the focal intercellular contact at the moment of transfer. Cii is the image in Ci rotated 45° around a central vertical axis in the direction shown. Images Bii, Ciii, and Dii are sectioned volume-rendered views; a sectioned plane is represented by a red box. (E–F) Shuttle demonstrating tethering of the departing recipient macrophage following a shuttle. Panel E presentation organized as in panel A. The tethered moment of transfer is detailed by volume-rendering in F, presented as in panels B–D. Scales as shown. Stills in A and E correspond to S1A and S1B Movie, respectively. *Eco*.*Nfsb*, *Escherichia coli* nitroreductase; EGFP, enhanced green fluorescent protein; *Gal4FF*, engineered form of *Saccharomyces cerevisiae* Gal4 transcriptional activator; *mpeg1*, macrophage-expressed gene 1; *mpx*, myeloid-specific peroxidase; *Tg*, transgenic; *UAS-E1b*, upstream activating sequence fused to minimal adenovirus E1b promoter.

While studying leukocyte behavior during the establishment of *T*. *marneffei* infection following inoculation of live conidia into zebrafish [20], we unexpectedly observed the recurrent direct transfer of conidia from live neutrophils to adjacent live macrophages (Fig 1; S1A and S1B Movie). The phenomenon was revealed by combining a 3-color fluorescent reporter system (labeling neutrophils in green with enhanced green fluorescent protein [EGFP], macrophages in red [mCherry], and conidia in blue [calcofluor]) with high-spatiotemporal–resolution live confocal imaging. We called this new form of interphagocyte pathogen transfer “shuttling.” Two defining features of shuttling distinguished it from other previously described forms of pathogen transfer. Firstly, shuttling occurred between live leukocytes, demonstrated by the mobility of both donor neutrophil and recipient macrophage before, during, and after shuttles. Secondly, the dynamic morphology of shuttling suggested purposeful rather than random exchange through a tethered cell-to-cell contact.

To characterize the dynamic morphology of shuttling comprehensively, we systematically collected multiple unselected examples from extensive confocal live-imaging microscopy experiments. To ensure that shuttles were unequivocally distinguished from all other modes of intercellular pathogen transfer, stringent criteria were applied for events to be included in this initial panel. For inclusion as a shuttle, all three phases of donation, transfer, and receipt were required to be unequivocally visualized (see Materials and methods for full details). The resulting collection of unequivocal shuttles comprised 13 examples of live *T*. *marneffei* conidial shuttling (S1 Fig), and as shuttling mechanisms were explored, another 17 unequivocal examples of conidial shuttling and 18 examples of the shuttling of other particles meeting all stringent definition criteria were collected (S1 Table).

Shuttling of live *T*. *marneffei* conidia occurred only in the first 2 h of infection establishment (median time of shuttle, 33 min [range 14–97] from commencement of imaging; *n* = 13 shuttles collected in 69 movies; S1A Fig). In contrast, no *T*. *marneffei* shuttles occurred during >181 h of imaging after 2 h postinoculation.

**Fig 2:**
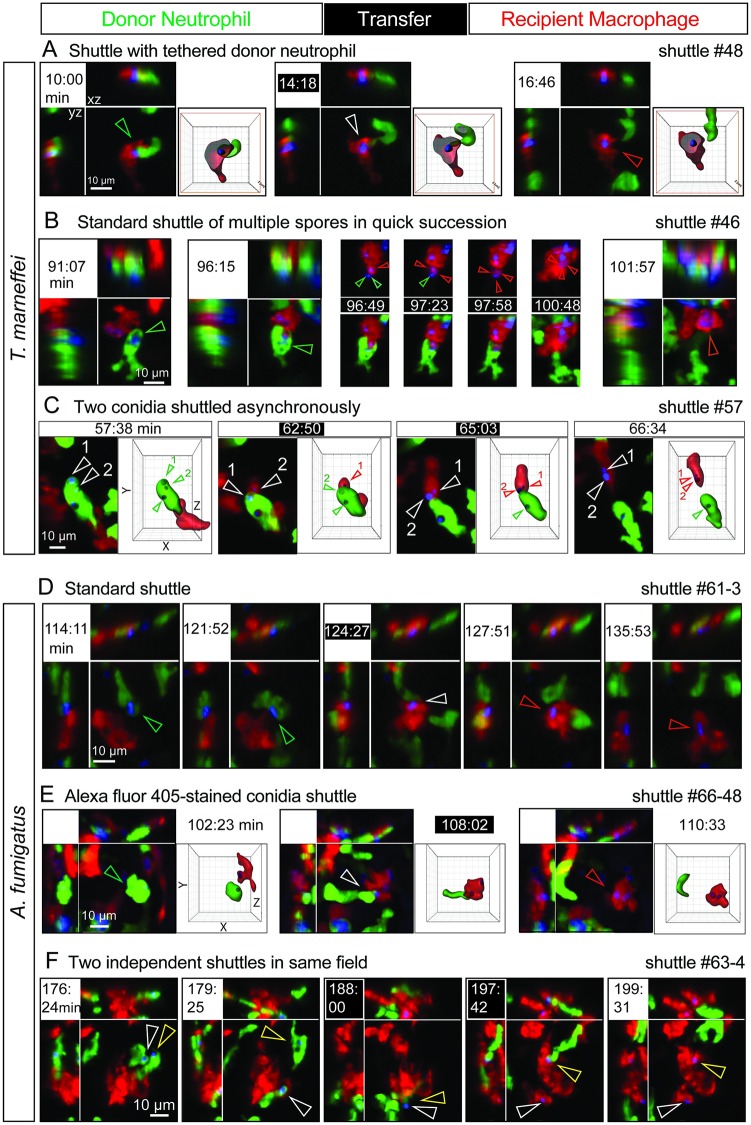
Variant shuttles of fungal conidia from neutrophil to macrophage. A variety of shuttles of conidia (blue) from *Tg(mpx*:*EGFP)* neutrophils (green) to *Tg(mpeg1*:*Gal4FF)×(UAS-E1b*:*Eco*.*NfsB-mCherry)* macrophages (red). In each example, panels include isometric orthogonal *yz* and *xz* views corresponding to the *xy* maximal intensity projection and indicate the time in min from start of movie. Time points colored white-on-black are the moments of transfer. (A,C,E) include volume-rendered views corresponding to the maximal intensity projection; where this volume is sectioned (as in panel A), the framing box is shown in red. Colored arrowheads indicate the conidium within donor neutrophil (green), at the point of intercellular transfer (white), and in the recipient macrophage (red). (A–C) Shuttles of *T*. *marneffei* conidia. (A) Shuttle demonstrating tethering of the donor neutrophil at the moment of transfer. (B) Shuttle of multiple conidia from one donor neutrophil in quick succession. First two frames show donor neutrophil laden with multiple conidia at two preshuttle time points. Frames from *t* = 96:49–100:48 min are maximal intensity projections only, encompassing the shuttling transfer of 3 conidia over 4 min Upper row of panels shows red (macrophage) and blue (conidia) channels only; lower row of panels includes the green channel (donor neutrophil). (C) Shuttle of 2 conidia from one donor neutrophil one after the other at an interval of 2 min 13 s. Volume-rendered images corresponding to maximal intensity projections show the 2 shuttled conidia (labeled 1 and 2) before, during, and after the shuttle. (D–F) Shuttles of *A*. *fumigatus* conidia. (D) Standard shuttle of single conidium. (E) Standard shuttle of conidium labeled with Alexa Fluor 405 rather than calcofluor, accompanied by volume-rendered images that focus attention onto the conidium and donor neutrophil of interest. (F) Two independent shuttles by different donor neutrophils occurring in the same field. In this series, the course of each shuttled spore is followed by white and yellow arrowheads. Scales as shown. Stills in A–F correspond to S1C, S1E, S1F, S2A, S2C and S2E Movies, respectively. *Eco*.*Nfsb*, *E*. *coli* nitroreductase; EGFP, enhanced green fluorescent protein; *Gal4FF*, engineered form of *S*. *cerevisiae* Gal4 transcriptional activator; *mpeg1*, macrophage-expressed gene 1; *mpx*, myeloid-specific peroxidase; *Tg*, transgenic; *UAS-E1b*, upstream activating sequence fused to minimal adenovirus E1b promoter.

These *T*. *marneffei* shuttling examples exhibited morphological features with mechanistic implications. In several cases, the donor neutrophil and/or recipient macrophage formed a highly polarized shape resulting from cell-to-cell tethering around the time of shuttling (Figs 1A, 1F and 2A; S1B–S1D Movie). These drawn-out tethered extensions of neutrophil and macrophage cytoplasm before, during, or after shuttling indicated a focal rather than a whole-of cell “hugging” interaction between them. Furthermore, this tethering confirms that the cells come into direct physical contact for the shuttle rather than merely moving into close proximity and transferring the conidium by expulsion into the extracellular space and rephagocytosis.

Although single conidia were usually shuttled (Fig 1, S1 Table), occasionally more than one conidium was transferred (2/13 instances; Fig 2B and 2C; S1E–S1F Movie). One example of this was in quick succession (Fig 2B, S1E Movie). However, the nonsynchronous transfer of 2 shuttled conidia in series from the same donor neutrophil in another example (Fig 2C, S1F Movie) indicated that the signaling mechanism driving each shuttle could operate independently.

### Shuttling also occurs with *A*. *fumigatus* conidia

To test whether conidial shuttling was specific to *T*. *marneffei* or a general phenomenon of fungal infection establishment, we assayed for shuttling following inoculation with live *A*. *fumigatus* conidia, another fungus whose interactions with leukocytes are well studied in zebrafish models [20–23] but for which shuttling has not previously been described. Seven unequivocal shuttles of live *A*. *fumigatus* conidia occurred in 6/22 imaging sequences (Fig 2D and 2F, S1B Fig, S2 Movie). The median time of shuttling was 121 min (range 30–199) following commencement of imaging. *A*. *fumigatus* shuttles exhibited similar features to *T*. *marneffei* shuttles, including cell-to-cell tethering (S2D Movie). In 1/7 examples, 2 shuttles occurred in the same imaged volume between different donor neutrophils and macrophages, separated by an interval of 10 min (Fig 2F).

To exclude the possibility that shuttling was an artifact of labeling conidia with calcofluor, we tested conidia with an alternate label. *A*. *fumigatus* conidia labeled with Alexa Fluor 405 were also shuttled (Fig 2E, S2C Movie). All shuttles occurred from donor neutrophil to recipient macrophage. No macrophage-to-neutrophil shuttles were observed despite looking carefully for them.

Collectively, these observations establish that shuttling is a recurrent form of unidirectional pathogen transfer from neutrophils to macrophages that occurs early in fungal infection establishment. It is not a peculiarity of the host response to a particular fungal pathogen because it occurs with two fungal species.

### Incidence of shuttling

Shuttling events meeting our stringent criteria were observed in 20/91 (22%) unselected imaging sequences of >60 min duration (S1 Fig). While this ascertainment rate provided a scorable surrogate categorical variable for shuttling incidence, a more biologically relevant measure of the incidence of shuttling would add weight to its biological significance.

One such biologically relevant quantification is the shuttling incidence per conidium at risk of shuttling. This measure, regardless of any macrophage phagocytic activity and recruitment of nonphagocytosing leukocytes, is denominated solely by the number of spore-laden neutrophils in the imaged volume available to act as donors. While this is impossible to determine exactly for any single image series because of neutrophil flux through the imaged volume, it is possible to compute an averaged estimate. For both these fungi, we have previously examined phagocytosis during infection establishment and previously reported that macrophage phagocytosis predominates over neutrophil phagocytosis in the first 3 h following inoculation [20]. These phagocytosis data resulted from analysis of a subset of imaging files of the current dataset and so provide a basis for estimating an averaged shuttling incidence based on averaged spore-laden neutrophil phagocytosis rates. For *T*. *marneffei*, an average of 1.34 conidia-loaded neutrophils (67 neutrophils at 50 time points) were present at any time in the imaged volume to be available as donors throughout the first 180 min after inoculation (derived from *n* = 10 imaging series, being those 10 series closest to 180 min in length). Hence, 13 shuttles in 69 imaging series means that on average, 14% of spores available in neutrophils for donation were shuttled in 3 h. Also of note is the fact that 5/10 imaging series had only ≤1 spore-laden neutrophil present in the imaged volume during the first 180 min, and hence, these imaging series provided little opportunity for shuttling to occur. For *A*. *fumigatus*, neutrophil phagocytosis of conidia was much rarer, as also observed by others [20,21]. An analogous averaged calculation gives an average incidence of 44% for shuttling of *A*. *fumigatus* spores that were available for donation by loaded neutrophils in the 3 h after inoculation (36 neutrophils/50 time points = 0.72 spore-laden neutrophils on average at any time point over 3 h; 7 shuttles in 22 movies).

Calculating the real shuttle incidence is challenging, and these rates are certainly underestimates. The rate depends on the sensitivity of ascertainment, which is constrained by the limitations of the detection method and our stringent definition of shuttling. These two factors together conspire to underestimate shuttle incidence.

Challenges in shuttle detection that contributed to underestimating incidence included (1) shuttles can currently only be recognized by the laborious method of manually observing them in retrospective analysis of imaging datasets; (2) at the magnification required for the subcellular resolution needed to see shuttles, the imaged volume is only a small fraction of the infected volume; and (3) the leukocytes involved are highly mobile and frequently move out of the imaged volume, and hence, the denominators for computing incidence are constantly changing.

**Fig 3:**
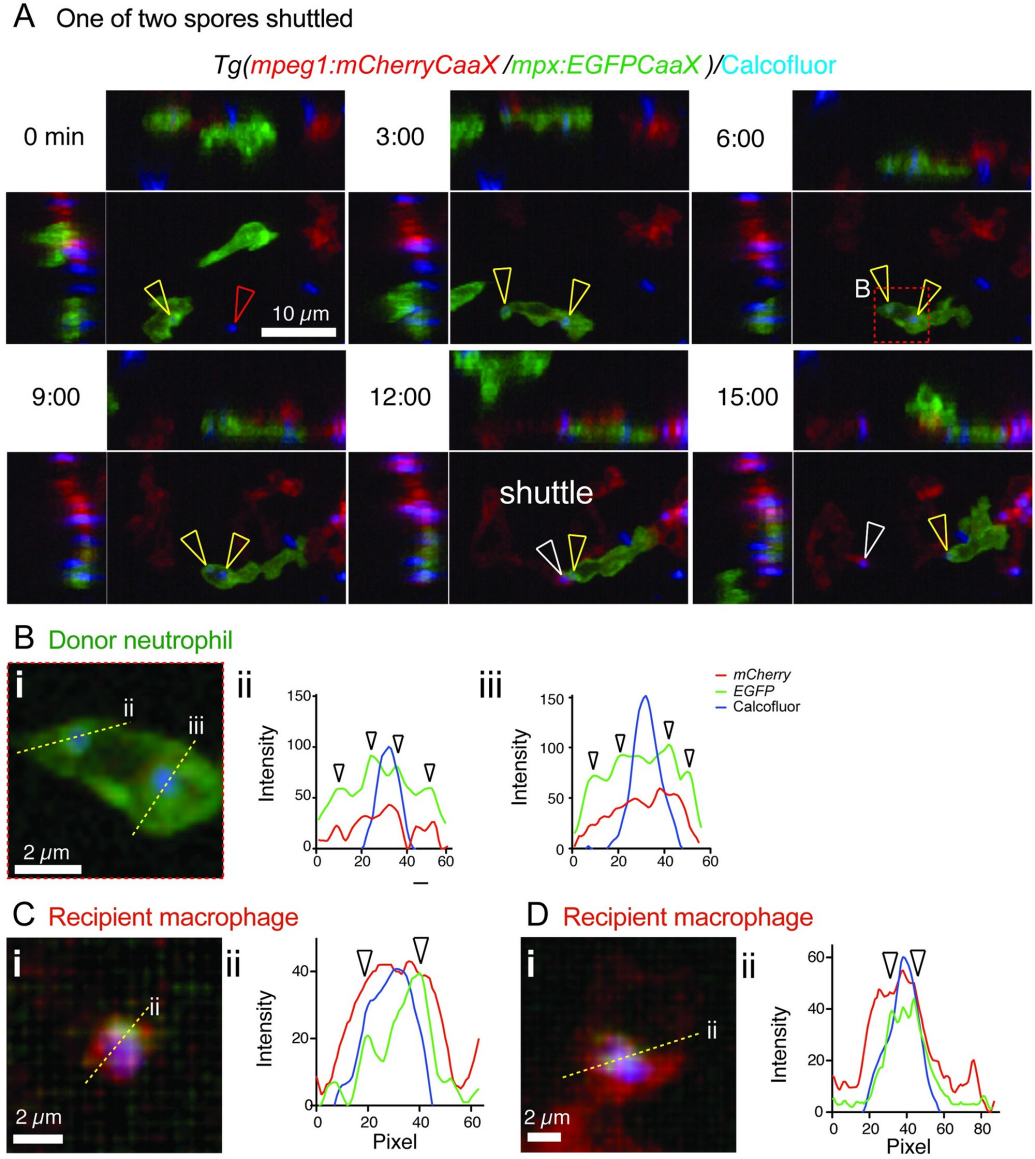
Shuttling of *T*. *marneffei* conidia between neutrophils and macrophages involves phagosome transfer. (A) Shuttle of calcofluor-stained conidium (blue) from *Tg(mpx*:*EGFP-CaaX)* neutrophils (green) to *Tg(mpeg1*:*mCherry-CaaX)* macrophages (red). These reporter lines have membrane-localized fluorophore expression. Panels include isometric orthogonal *yz* and *xz* views corresponding to the *xy* maximal intensity projection and indicate the time in min from start of the movie. Colored arrowheads indicate a conidium before it is phagocytosed by the donor neutrophil (red), conidia within the donor neutrophil (yellow), and the conidium at the point of intercellular transfer and within the recipient macrophage (white). (Bi) Detail of the boxed area of the donor neutrophil in (A), 6-min panel. Yellow dotted line indicates the position of the cross-section for the 3-color fluorescence intensity plots in (ii) and (iii). Both shuttled and nonshuttled conidia are flanked by peaks of green fluorescence, consistent with their location in a membrane-lined phagosome. (C,D) Cross-sections fluorescence intensity profiles (ii) corresponding to the yellow lines in (i) for 2 macrophages that received a spore from a neutrophil in this dataset, which contained 3 independent spore shuttles. The arrowed EGFP-channel signal demonstrates the transfer of neutrophil-derived EGFP-tagged membrane in the vicinity of the spore (blue channel signal). Scales as shown. Stills in A correspond to S3A Movie. EGFP, enhanced green fluorescent protein; *mpeg1*, macrophage-expressed gene 1; *mpx*, myeloid-specific peroxidase; *Tg*, transgenic.

Several other observations indicate that shuttles are not rare. If shuttles were rare, it would be unlikely that multiple examples would occur together or in the same imaging sequence. However, 5/20 datasets contained examples of multiple shuttles: either 2–3 spores being shuttled together or in quick succession or asynchronously from the same or several different donor neutrophils (Figs 2B, 2C, 2F and 3; S1E, S2F, S2E and S3A–S3C Movies).

The stringent criteria applied to ensure only unequivocal shuttles were included also means that the shuttle incidence is likely to be underestimated. Multiple events that were probably shuttles were excluded from this initial panel of unequivocal shuttles (see examples in S2 Fig, S4 Movie). There were probable shuttles for which the conidium could not unequivocally be resolved as within the donor neutrophil rather than adherent to it (S2A Fig, S4A Movie). The criterion most often not met was clear visualization of the donor–recipient cell-to-cell contact at the point of conidial transfer (S2C and S2D Fig, S4C–S4D Movie). This scenario included instances in which a phagocytosed particle appeared to be deposited by the neutrophil into extracellular space and was then subsequently taken up by a macrophage. In some imaged volumes, a large number of highly active neutrophils and macrophages were attracted to the inoculated spores, and it was impossible to separate what was happening, although many spores were initially within neutrophils that ended up in macrophages (S2D Fig, S4D Movie). All imaging datasets collected for these studies have been included in the denominator of our unselected series despite such features (S1 Fig, S1 Table).

From these data and considerations, we conclude that although the detection of shuttling is laborious and challenging, shuttling itself is not a rare phenomenon. For both these fungal pathogens, those spores that are initially phagocytosed by neutrophils have a substantial chance of being shuttled to macrophages in the first 3 h of infection establishment.

### Shuttling involves phagosome transfer

We previously reported the transfer of neutrophil cytoplasm to macrophages in the context of inflammation [24]. We therefore hypothesized that shuttling could also involve transfer of donor neutrophil cytoplasmic components to the recipient macrophage. To test specifically whether neutrophil membrane was also transferred, we imaged shuttling in transgenic (*Tg*) macrophage-expressed gene 1 (*mpeg1*):*mCherry-CaaX/*myeloid-specific peroxidase (*mpx*):*EGFP-CaaX)* embryos, in which the fluorescent labeling of neutrophils and macrophages is localized to the membrane via a prenylation motif (Fig 3, S3 Movie). Cross-sectional fluorescence intensity profiling of conidia in these transgenic lines demonstrated that prior to shuttling, about-to-be shuttled conidia reside within membrane-bound compartments within the neutrophil (Fig 3B). Observation of shuttled conidia in macrophages immediately following transfer demonstrated that green fluorescent signal surrounding the spore, attributable to the neutrophil membrane, was also transferred to the macrophage (Fig 3C).

This provides direct evidence that shuttled conidia are located in a membrane-lined subcellular neutrophil compartment, likely to be a neutrophil phagosome, that is shuttled in its entirety to the recipient macrophage. The rapid decay of the cytoplasmic neutrophil reporter fluorophore signal following shuttling suggests that within the macrophage it is either quenched because of pH change or that the structure of the shuttled phagosome and its component proteins are rapidly destroyed by the macrophage. Further evidence that shuttled particles reside within the acidified phagolysosome environment of donor neutrophils before shuttling and recipient macrophages after shuttling was generated from an in vitro assay using murine leukocytes (see below).

### Phagocyte motility confirms that living cells participate in shuttling

Our previous studies demonstrated that phagocytes exhibit lineage- and site-specific spatiotemporal responses during establishment of fungal infection [20]. We asked whether shuttling occurred “on the fly” between fast-moving cells or whether cells slowed down and “parked” to engage in this intercellular interaction.

**Fig 4:**
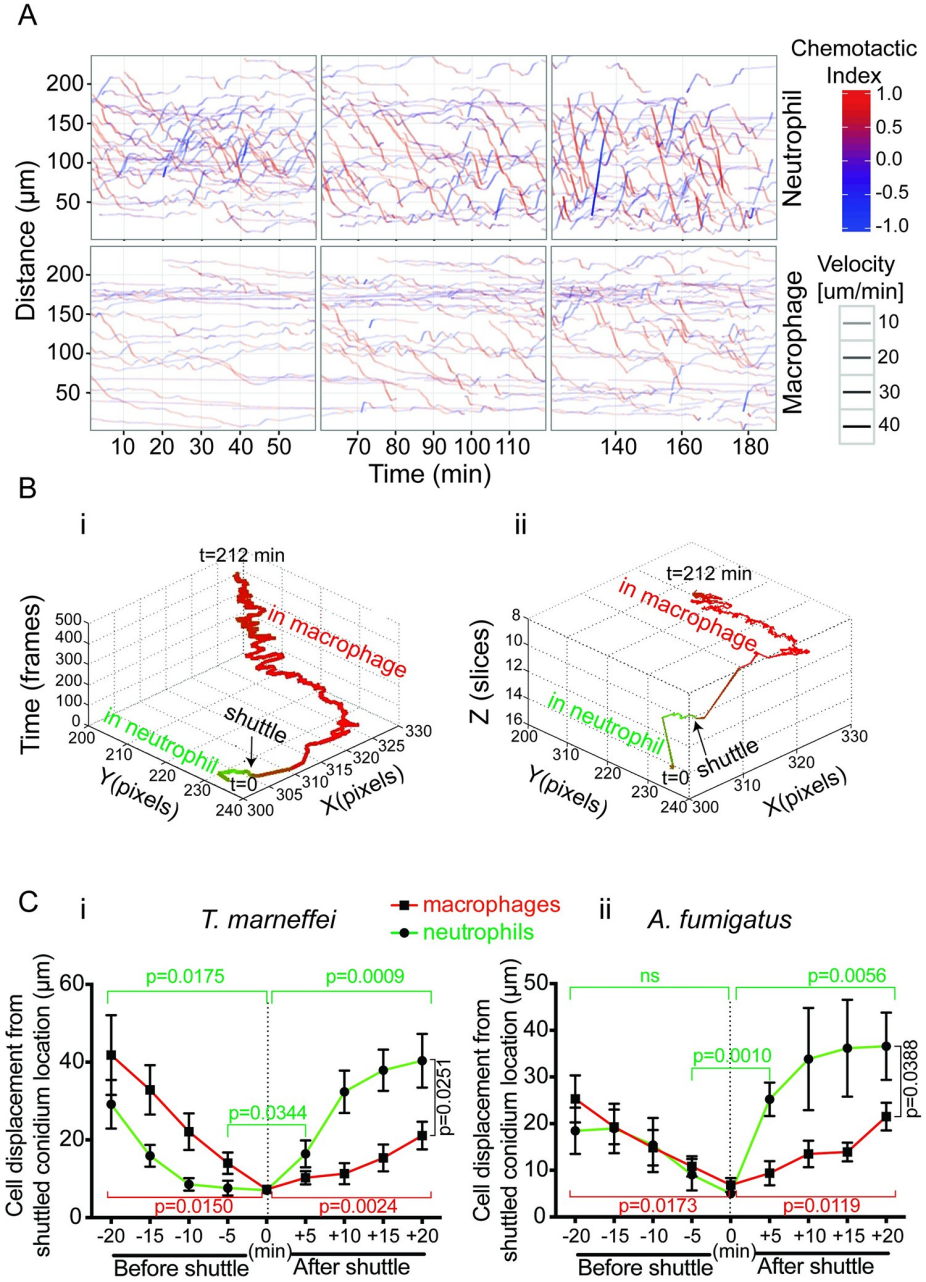
Phagocyte mobility during shuttling. (A) Cell tracking analysis of neutrophil and macrophage following intramuscular inoculation of *T*. *marneffei* conidia. A chemotactic index (red, movement towards infection; blue, movement away) and velocity (thickness of the line). Neutrophil migration towards the infection is earlier and faster; macrophage migration is later and slower. (B) Output of ShuttleFinder software for the shuttle shown in Fig 1A. Track color indicates the cellular context of the conidium (green, neutrophil; red, macrophage). (i) shows the shuttled spore track in *xy* dimensions and time. (ii) shows it in *xyz* dimensions. The two outputs collectively show that the shuttle occurred between donor and recipient phagocytes that were mobile in both space and time. Imaging parameters: *x*, *y*, 1.31 pixels/μm; *z* interval, 3.3 μm/slice; frame rate, 28.0 s/frame. (C) Plots of donor neutrophil and recipient macrophage cell displacement from the shuttle spore location over the 20-min period before and after the moment of shuttle for *T*. *marneffei* (i) and *A*. *fumigatus* (ii) shuttles. Note that displacement is a measured distance without directional information. Data are mean ± SEM at each time point (i, *n* = 10; ii, *n* = 7). *P*-values from two-tailed paired t tests (all +20 and -20 min vs 0 comparisons), otherwise from one-tailed paired t tests. Datasets for C are provided in S1 Data. ns, not significant.

We first focused on the scenario in which *T*. *marneffei* conidia were delivered into the somite. To characterize the overall picture of leukocyte movement in which shuttling occurred, we used 4-dimensional cell tracking in Imaris software (Bitplane; Andor Technology, Belfast, UK) to extract and plot cell coordinates in time and space. We interrogated these data using the open source programming language R as has been used to analyze leukocyte swarming [25]. In this scenario, neutrophils started to migrate towards the infection site soon after inoculation with conidia, while macrophage migration initiated later, during the second hour postinfection. Overall, neutrophils exhibited more rapid motility than macrophages at all times and in all directions (*p* < 0.0001). Phagocytosis of conidia upon arrival at the site of infection was associated with a reduction in migration velocity for both neutrophils and macrophages (Fig 4A).

To focus on the subset of phagocytes engaging in shuttling within this melee of phagocyte activity, we developed “ShuttleFinder,” a MATLAB (The MathWorks, Natick, MA, USA) program based on “PhagoSight” [26] that performs spatiotemporal tracking of conidia and reports the color of their immediate surrounding environment (Fig 4B). ShuttleFinder did not facilitate automatic shuttle discovery because of the high number of disjointed tracks and a high number of false positives. However, it enabled the paths of conidia that were shuttled to be displayed in 2-dimensional space and time (Fig 4Bi) and 3-dimensional space (Fig 4Bii). This demonstration of conidial translocation showed the extent to which the donor neutrophils and recipient macrophages in which shuttled spores resided were mobile prior to and following the shuttle (Fig 4B and 4C).

We next assessed whether cell velocity changed during shuttling by manually examining the displacement of neutrophils and macrophages relative to the shuttle site over fixed 5-min intervals for the 20-min period before and after shuttles. Neutrophil displacement was significantly less for the 5-min period immediately before a shuttle than the period just after shuttling, indicating a faster neutrophil directional velocity immediately following shuttling (Fig 4C). Furthermore, neutrophil displacement from the shuttle site by 20 min after shuttling was at least as great as that at 20 min before, confirming that shuttling neutrophils remained as actively mobile as before (Fig 4C). The ongoing movement of neutrophils after shuttling strongly indicates that the donor neutrophil remained alive.

Macrophages also moved towards and away from the shuttle point, confirming their viability (Fig 4C). As expected, for shuttles of both conidial types, macrophage average directional velocity over the 20 min after shuttling was significantly slower than that of the departing neutrophils (*T*. *marneffei*: 1.1 ± 0.5 versus 2.0 ± 1.2 μm/min, *p* = 0.0251; *A*. *fumigatus*: 1.1 ± 0.4 versus 1.8 ± 1.0 μm/min, *p* = 0.0388; one-tailed *t* test) (Fig 4C). Over longer periods of time, recipient macrophages were highly mobile (Fig 4B), also confirming their ongoing viability throughout the shuttling process.

**Fig 5:**
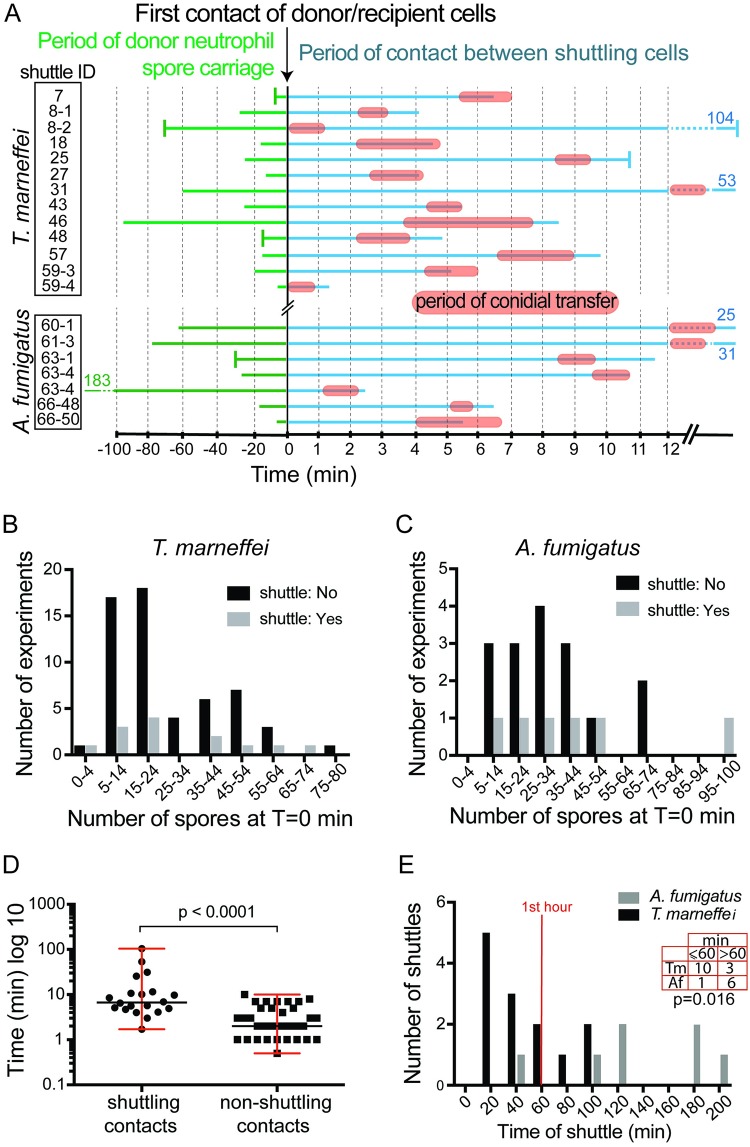
Dynamics of neutrophil-to-macrophage conidial shuttles. (A) Time maps of cellular contact during shuttles. Charts are aligned with *t* = 0 at the point of initial donor–recipient cell contact. Chart shows time of conidial residence in donor neutrophil prior to intercellular contact (green line; vertical bar indicates resident at start of imaging; otherwise, line start indicates point of neutrophil phagocytosis), time of general intercellular contact (blue line), and period of contact during actual conidial transfer (orange bars). Charts are for each of 20 stringently defined unequivocal shuttles (*n* = 13 *T*. *marneffei*, *n* = 7 *A*. *fumigatus*) tabulated and identified as in S1 Fig. The entire sequence from phagocytosis to cell separation is shown unless vertical bars at beginning or end of lines indicate the start or end of the imaging file; blue and green numbers indicate endpoint times where lines have been clipped. (B,C) For *T*. *marneffei* (B) and A. *fumigatus* (C) infections, histograms of number of experiments with and without observed shuttles are shown by number of conidia present in the initial imaging volume. (D) For conidia-laden neutrophils, duration of shuttling contacts (*n* = 20 shuttles) compared to random nonshuttling contact (*n* = 34) is shown. Data are summarized by median and range. (E) Distribution of times of shuttle after imaging commenced for *T*. *marneffei* and *A*. *fumigatus*. *p* = 0.016 for categorical variable of shuttles occurring at ≤60 and >60 min between the two fungal species (Fisher’s exact test). Datasets for A–E are provided in S1 Data.

The 20 shuttles of live spores meeting all stringent definition criteria (Fig 5A) suggested that shuttling continues the process of conidial dissemination. In 16/20 cases, the donor neutrophil entered the field during the imaging period, and hence, it had picked up its spore for donation elsewhere. Furthermore, in 18/20 cases, the recipient macrophage separated from the donor neutrophil before the image sequence finished (Fig 5A).

In summary, these data show that neutrophils slow down to donate conidia for shuttling and speed up on departing, remaining viable throughout the process. The recipient macrophages also remain actively mobile throughout the shuttling process. The mobility of shuttling cells before and after shuttling contributes to the ongoing process of spore dissemination during infection establishment.

### Shuttling is a purposeful interaction that involves sustained intercellular communication

We considered the possibility shuttling might be a chance event rather than purposeful interaction. If shuttling occurred by chance alone, then the number of shuttles would be expected to be a function of the number of conidia delivered. However, shuttles occurred in fields that had initially as few as <4 conidia and as many as 75–100 conidia for both fungal species, and we could not resolve a trend based on the number of conidia initially in the imaged volume (Fig 5B and 5C).

We also hypothesized that if shuttling were purposeful rather than a random event, then this would be reflected by purposeful intercellular interactions. Shuttling is characterized by multiple polarized interactions between the donor neutrophil and the recipient macrophage prior to the shuttle, indicating sustained and purposeful prior cell–cell communication (S1, S2 and S3 Movies). To quantitatively test the hypothesis that the degree of intercellular interaction between shuttling leukocytes was unusually extensive, we compared the duration of neutrophil–macrophage contacts that ended with a shuttle to randomly selected nonshuttling contacts. This analysis revealed that shuttling cells stay in contact for a significantly longer period prior to shuttling than is otherwise the case for random neutrophil–macrophage interactions (*p* < 0.0001) (Fig 5D). This observation is consistent with there being signals bringing the donor and recipient cells together prior to shuttling. Furthermore, it indicates that these signals are different from those that attracted the leukocytes to migrate to the site of infection.

### Pathogen-dependent shuttling kinetics indicates a conidial determinant

We hypothesized that the molecular mechanism driving shuttling likely involved fungal determinants. If this were the case, this might result in different kinetics for the shuttling of conidia of different fungal species. Although we observed no obvious morphological difference between shuttles of *T*. *marneffei* and *A*. *fumigatus* conidia (suggesting that the mechanism driving shuttling is fundamentally the same for both), the kinetics of shuttling events differed for the two species. *T*. *marneffei* shuttles occurred predominantly in the first hour after inoculation, whereas *A*. *fumigatus* shuttles happened at later time points (*p* = 0.016) (Fig 5E). There was no ascertainment bias for shuttles of one or other fungus because the 2 movie datasets shared a similar distribution of imaging durations (S1 Fig; the two distributions of movie lengths are not significantly different (*p* = 0.1985, Mann–Whitney U test)).

This important observation indicates that although shuttling is a general phenomenon in fungal infection establishment, because the kinetics of shuttling is specific to the fungal species, determinants of the molecular mechanism reside in properties of the conidia themselves.

### The fungal determinant of shuttling is not dependent on conidial viability

**Fig 6:**
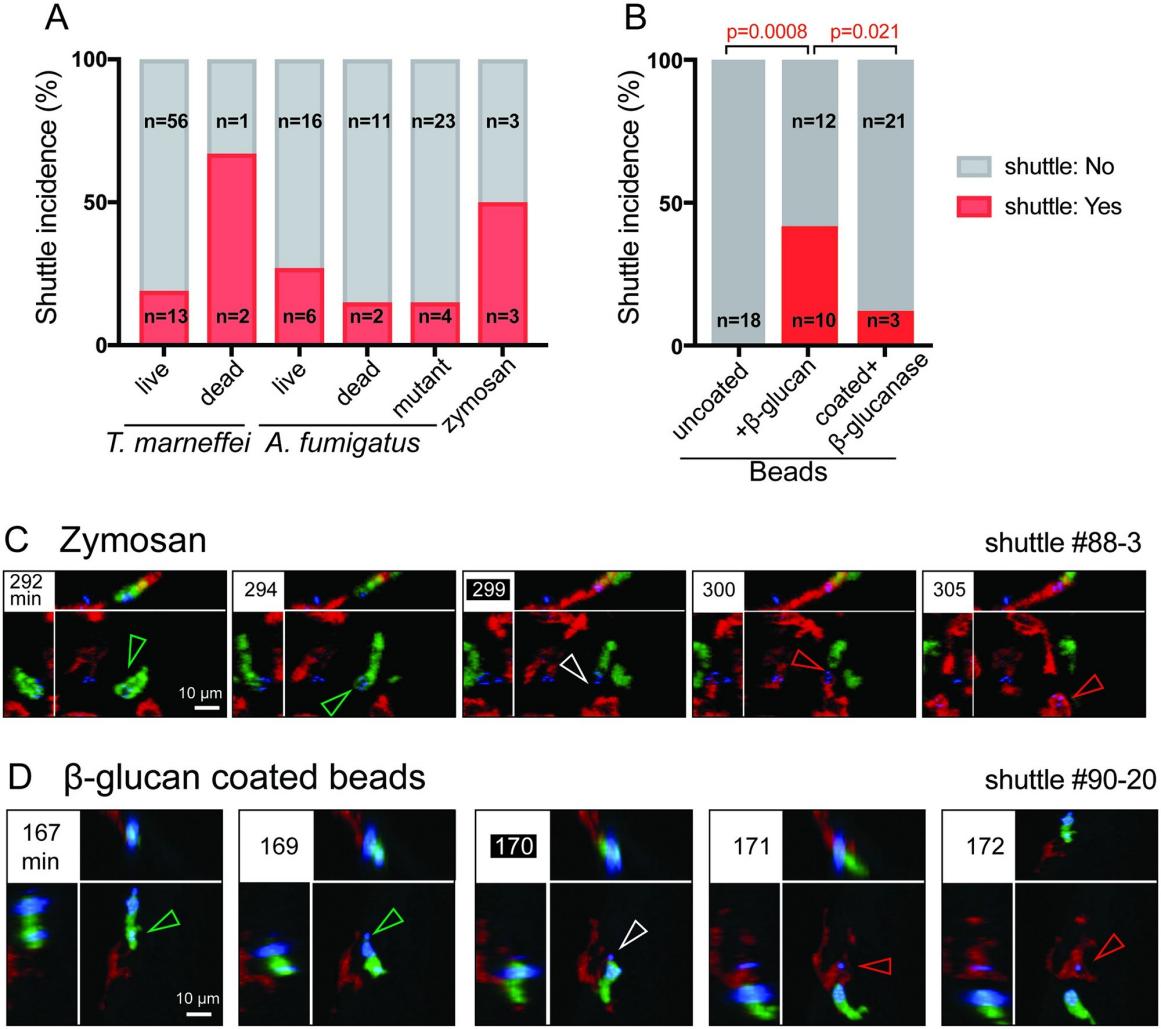
β-glucan is a fungal determinant sufficient to trigger shuttling. (A,B) Relative frequency of shuttles for different cargos, incidence computed for each condition as number of 3-h imaging datasets with shuttle(s)/total number of imaging datasets. By chi-squared analysis, there are no significant differences for the comparisons: live spores of the two species (*p* = 0.38), live and dead *T*. *marneffei* (*p* = 0.31), and dead spores of the two species (*p* = 0.34). *n*-values indicate the number of datasets in each category. (C,D) Images of representative shuttles of zymosan particle (C) and β-glucan–coated plastic beads (D). Shuttles of particles (blue) are from *Tg(mpx*:*EGFP)* neutrophils (green) to *Tg(mpeg1*:*Gal4FF)×(UAS-E1b*:*Eco*.*NfsB-mCherry)* macrophages (red). In each example, panels include isometric orthogonal *yz* and *xz* views corresponding to the *xy* maximal intensity projection and indicate the time in min from start of movie. Colored arrowheads indicate the conidium within donor neutrophil (green), at the point of intercellular transfer (white) and in the recipient macrophage (red). Scales as shown. Stills in C, D correspond to S5A and S5C Movie, respectively. Datasets for A–B are provided in S1 Data. *Eco*.*Nfsb*, *E*. *coli* nitroreductase; EGFP, enhanced green fluorescent protein; *Gal4FF*, engineered form of *S*. *cerevisiae* Gal4 transcriptional activator; *mpeg1*, macrophage-expressed gene 1; *mpx*, myeloid-specific peroxidase; *Tg*, transgenic; *UAS-E1b*, upstream activating sequence fused to minimal adenovirus E1b promoter.

A fungal determinant of shuttling could be either a chemical constituent of the conidium or a newly synthesized metabolite of the germinating fungi. To distinguish between these possibilities, we microinjected conidia inactivated by either freezing (*T*. *marneffei*) or γ-irradiation (*A*. *fumigatus*) and imaged for shuttling events. We observed multiple shuttles of inactivated fungal conidia (Figs 3 and 6A; S3 Movie), confirming that shuttling was stimulated by non-temperature–labile components of the conidium rather than a newly synthesized signal.

### β-glucan is a fungal determinant sufficient for shuttling

Because shuttling was independent of conidial viability, we hypothesized that the spore-derived signal for shuttling was either from the shape or size of the particle or was a chemical component of the fungal cell wall such as chitin or β-glucan.

To test whether particle size was sufficient to trigger shuttling, we microinjected 1.7- to 2.2-μm fluorescent particles (approximating the size of *T*. *marneffei* and *A*. *fumigatus* conidia) into the tail somite of 2–3 days postfertilization (dpf) zebrafish embryos. Although the beads were actively phagocytosed by both neutrophils and macrophages, no shuttling events were observed (approximately 60 h of imaging, 19 experiments) (Fig 6B). During these experiments, we frequently observed efferocytosis of entire bead-laden neutrophils by macrophages (S3 Fig, S5B Movie). While the neutrophil EGFP fluorescent signal was rapidly lost following engulfment, the bead-conjugated fluorophore signal persisted. These experiments determined that being a particle of a particular size was not sufficient to induce shuttling and that leukocyte phagocytic behavior towards inert beads was demonstrably different to their response to fungal conidia. This indicates that shuttling is a behavior driven at least in part by a chemical signal residing within the conidium itself.

The cell wall of fungal conidia is primarily composed of polysaccharides (chitin and glucans) and proteins [27]. The β-glucan class of polysaccharides are a major component of the conidial wall and are highly immunostimulatory, so they represented a promising candidate shuttling mediator. We confirmed that β-glucan was exposed on the surface of the ungerminated *T*. *marneffei* and *A*. *fumigatus* conidia as prepared as for shuttled inoculates (S4 Fig), making β-glucan a candidate fungal stimulus for shuttling. To test whether β-glucan was sufficient to induce shuttling, we first looked for shuttling of zymosan particles. Zymosan particles are approximately 3 μm in diameter and are a derivative of the *Saccharomyces cerevisiae* cell wall, a rich source of β-glucan glucose polymers. We observed 3 unequivocal shuttles from approximately 30 h of imaging over 6 experiments (Fig 6A and 6C). Because zymosan is predominantly β-glucan, these data suggested that β-glucan may be a spore-derived signal sufficient for shuttling.

To more rigorously test the ability of β-glucan itself to trigger shuttling, we tested whether coating plastic beads in β-glucan conferred on them the ability to be shuttled. While uncoated beads were not shuttled (0 shuttles in 19 experiments), beads coated with β-glucan were shuttled at relatively high frequency (10 shuttles during 22 experiments) (Fig 6B and 6D). Furthermore, treating β-glucan–coated beads with a mixture of β-glucanase enzymes (including endo/exo-1,3-β-D-glucanase and β-glucosidase) significantly reduced the rate of shuttle ascertainment from 10 shuttles in 22 experiments to 3 shuttles over 24 experiments (Fig 6B).

As a genetic model, we also tested Δ*gel1*Δ*gel7*Δ *A*. *fumigatus* α-glucosidase 1(*cwh41*) *A*. *fumigatus* spores, which are deficient in their cell-wall β-glucan content because of mutation of their *A*. *fumigatus* β-1,3-glucanosyltransferase (*gel*) genes (62.6% and 42% of wild type at 37 °C and 50 °C, respectively) [28]. The shuttle ascertainment rate for conidia from the mutant strain trended lower compared to wild-type *A*. *fumigatus* (27.3% in wild type versus 14.8% in mutant), but this difference was not statistically significant (Fig 6A), likely because the approximately 50% remaining β-glucan on the conidial cell wall remained sufficient to trigger shuttling.

Collectively, these data support the hypothesis that β-glucan is a fungal-wall–derived molecule that is sufficient to trigger shuttling signals.

### Shuttling also occurs between mammalian neutrophils and macrophages

Our studies in zebrafish revealed that shuttling was a conserved host response to different species of fungi. We also observed shuttling between murine neutrophils and macrophages, indicating that this behavior was conserved between phagocytes from different host species, including higher vertebrates such as mammals.

**Fig 7:**
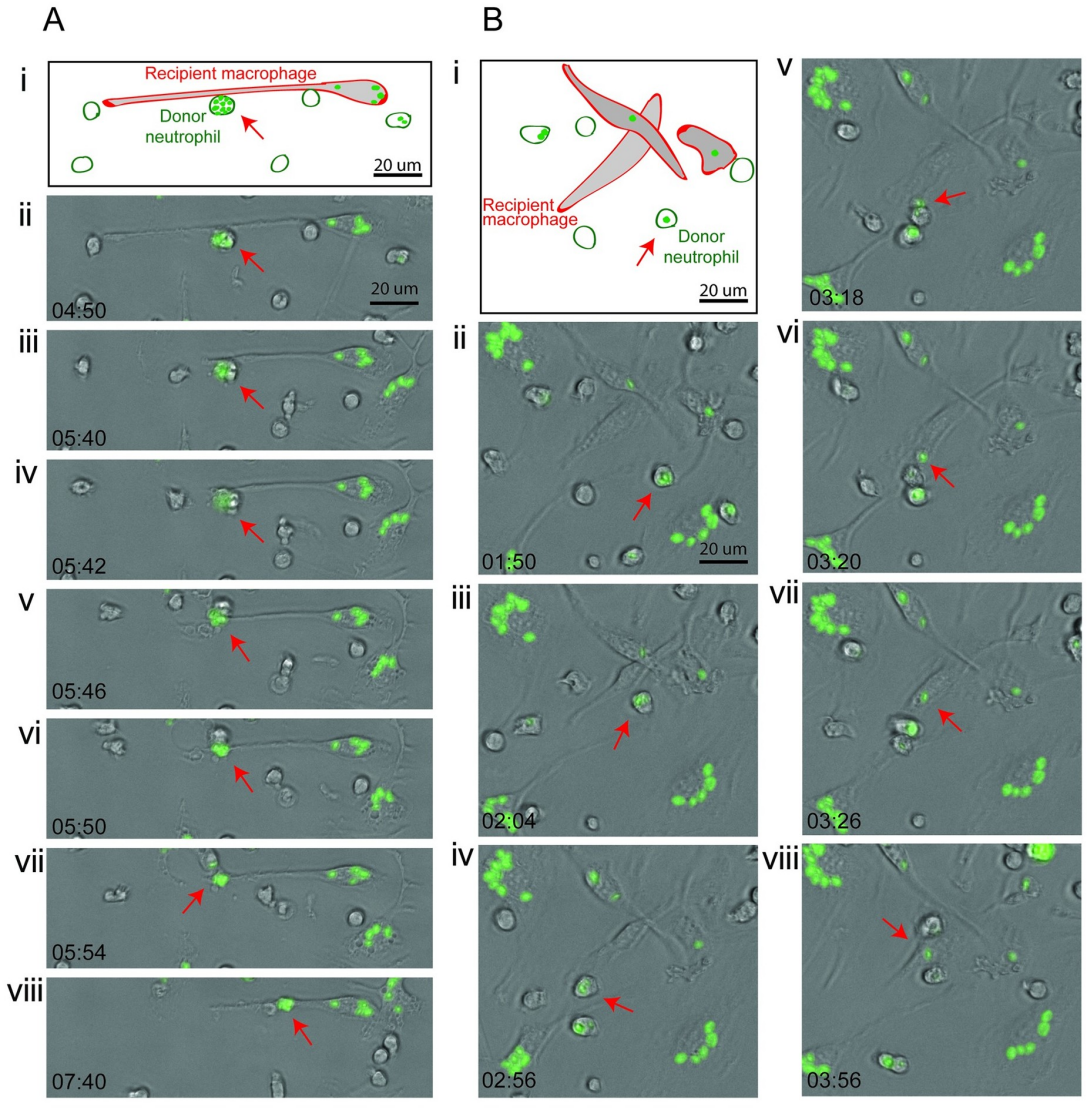
Zymosan shuttles by murine neutrophils and macrophages. (A,B) Two sequences demonstrating neutrophil-to-macrophage shuttling of Alexa-Fluor-488–labeled zymosan particle between murine phagocytes in vitro. Panel (i) is a schematic showing the elongated, adherent recipient macrophage. Panels (ii–viii) are bright-field photomicrographs with green fluorescence channel overlaid, with time points indicated in min:s. Red arrow indicates the shuttled particle in donor neutrophil (panels ii–vi) and then, following shuttling, within the recipient macrophage (panels vii–viii). Stills from S6 Movie.

Shuttling between murine phagocytes was observed in an in vitro assay. Primary mouse bone marrow neutrophils were preloaded with Alexa-Fluor-488–labeled zymosan added to mouse bone-marrow–derived macrophages and imaged over time. The transfer of zymosan particles from living neutrophils to macrophages was observed in a similar fashion to that observed in the zebrafish in vivo model (Fig 7A and 7B).

These data indicate that shuttling is a conserved behavior of phagocytes in vertebrates from zebrafish to higher mammalian models and is relevant to host–pathogen interactions during establishment of fungal infections in mammals.

**Fig 8:**
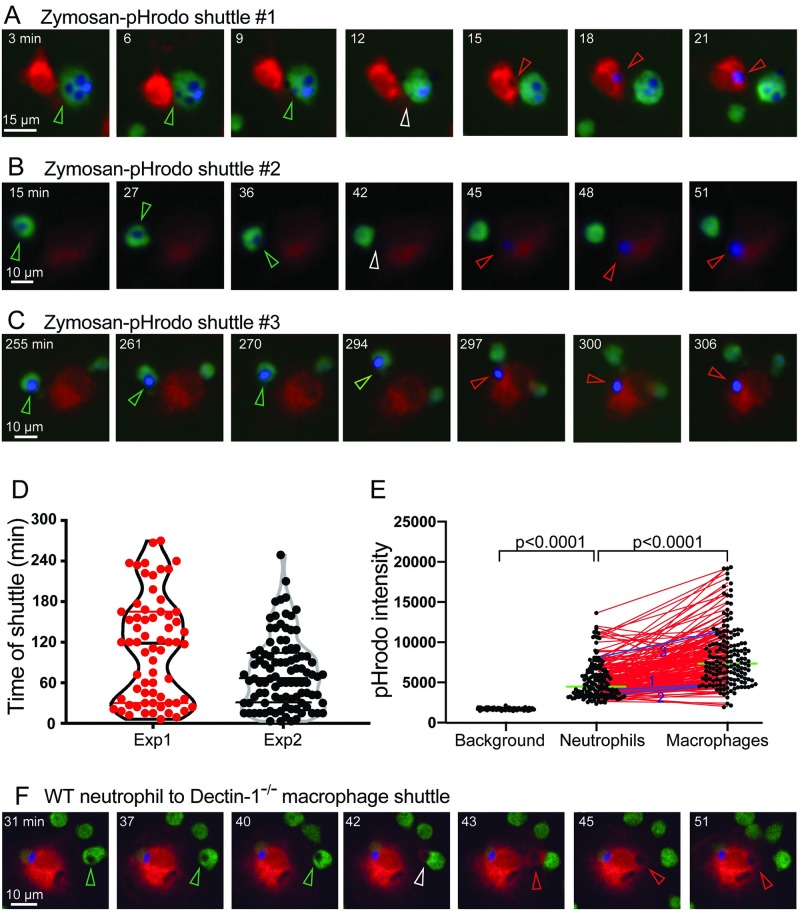
Shuttling of pHrodo-labeled zymosan particles between murine phagocytes in vitro. (A–C) Three examples of in vitro shuttling of zymosan–pHrodo particles, representative of 164 shuttling events. Neutrophils, green; macrophages, red; pHrodo–zymosan, false-colored blue. Scales as shown. Stills from S6C–S6E Movie. (D) Distribution of the time of shuttling for 164 in vitro shuttling events in panel C and S2 Table (Experiment 1, *n* = 66; Experiment 2, *n* = 98). (E) pHrodo signal intensity (arbitrary units) for in vitro shuttled zymosan–pHrodo particles (*n* = 164). Background intensity is for *n* = 100 randomly selected points clearly positioned between cells (*n* = 50 from each experiment). Black dots show the distributions of zymosan–pHrodo intensities in neutrophils 6 min before shuttling and in macrophages 6 min after shuttling; red lines connect paired values. *p*-values from unpaired two-tailed *t* test (background versus neutrophil) and paired two-tailed *t* test (neutrophil versus macrophage). Green line indicates median. Blue lines (1–3) correspond to the 3 examples shown in panels A–C. (F) Example of in vitro shuttling of a zymosan–pHrodo particle from a wild-type donor neutrophil to a Dectin-1^−/−^ recipient macrophage. Scale as shown. Stills from S6F Movie. Colored arrowheads (in A–C and D) indicate the conidium within donor neutrophil (green), at the point of intercellular transfer (white), and in the recipient macrophage (red). Datasets for D–E are provided in S1 Data. Exp, experiment; WT, wild type.

We employed this in vitro system to verify that shuttled particles were truly intracellular prior to and after their shuttling, using zymosan particles labeled with pHrodo, a dye that is nonfluorescent at neutral pH but fluoresces at acidic pH, as occurs in maturing phagosomes. The dataset comprises two independent 5-h imaging sequences capturing 3 fields per well, imaged at 3-min intervals, in which 164 shuttles were observed (Fig 8A–8D). Initially, there were many nonfluorescent intracellular particles, but progressively, all pHrodo-stained zymosan particles within neutrophils and macrophages fluoresced, indicating they were now within intracellular acidic environments (Fig 8A–8C and 8E; S6C–S6E Movie). Consistent with previous observations [29,30], a generally weaker pHrodo signal in neutrophils than macrophages indicated that the neutrophil phagosomes were less acidic than macrophage phagosomes (Fig 8A–8C and 8E). All 164 shuttled particles displayed pHrodo fluorescence in the donor neutrophil at a level significantly above background, indicating that before shuttling, they were within an acidified intracellular environment (Fig 8E, S6C–S6E Movie). Within the recipient macrophage, the shuttled particle remained in an acidic environment that became progressively more acidic, reflected by a progressively stronger pHrodo fluorescence signal (Fig 8E, S6C–S6E Movie). These data confirm that shuttled particles are not merely adhered to the donor cell but originate from within acidified donor neutrophil phagosomes. Furthermore, they are transferred to macrophage phagosomes that undergo further maturation with acidification.

The incidence of shuttling was determined for these in vitro assay conditions. At the densities of donor neutrophils, recipient macrophages, and zymosan particle loading rates used in these experiments, shuttling rates over 5 h were 4.8 and 20.0 shuttles per 100 loaded neutrophils (S2 Table). As the shuttles occurred through the entire 5-h ascertainment period (Fig 8D), this averages to 1 and 4 shuttles/100 loaded neutrophils/h. This provides further evidence that shuttling is a prevalent interaction between zymosan-laden neutrophils and macrophages.

Dectin-1 is considered the major β-glucan receptor in mammalian systems, although other cell-surface β-glucan–binding molecules also exist [31,32]. We therefore used our in vitro shuttling assay to test whether Dectin-1 expression was required for recipient macrophages to accept zymosan shuttles from neutrophils by using macrophages from Dectin-1 knockout mice. Shuttles from wild-type neutrophils to Dectin-1^−/−^ macrophages occurred, indicating that Dectin-1 is not absolutely required for zymosan shuttling (Fig 8F, S6F Movie). However, the incidence of shuttling was consistently lower when recipient macrophages were Dectin-1–deficient (Table 1). In two independent experiments, the shuttling rates with Dectin-1^−/−^ macrophages were 23% and 36% of the wild-type rates. The wild-type rates in these experiments (4.9 and 4.3 shuttles/100 loaded neutrophils at 20.4 min) corresponded well with the incidence data in S2 Table. These observations indicate that Dectin-1 is indeed involved in shuttling signaling, and there is a requirement for Dectin-1 for optimal shuttling efficiency.

**Table 1:** Zymosan–pHrodo shuttling involving Dectin-1–deficient recipient murine macrophages.

Experiment*	Shuttling Cell		Cell Number at Start* Fifth movie frame					Shuttles Mean ± SD	*p*-Value^†^ Wild type versus Dectin-1^−/−^
	Donor Neutrophils Genotype—source	Recipient Macrophages Genotype	Neutrophils Number/scored area		Macrophages Number/scored area		Number of Wells Scored		
			Loaded	Total	Loaded	Total			
1	wild type—BM	wild type	22	204	31	81	1	2	n/a
	wild type—BM	Dectin-1^−/−^	38	405	36	61	1	1	
	wild type—PB	wild type	18	203	41	114	1	1	n/a
	wild type—PB	Dectin-1^−/−^	15	248	34	90	1	0	
2	wild type—BM	wild type	356 ± 150	822 ± 251	60 ± 15	76 ± 17	3	17.3 ± 4.7	0.0115
	wild type—BM	Dectin-1^−/−^	418 ± 49	884 ± 135	47 ± 13	64 ± 16	3	4.7 ± 1.5	
3	wild type—BM	wild type	432 ± 84	891 ± 78	36 ± 14	54 ± 25	2	18.5 ± 3.5	0.0001
	wild type—BM	Dectin-1^−/−^	319 ± 62	661 ± 93	54 ± 15	80 ± 26	6	5.0 ± 1.2	

*Experimental conditions are detailed as follows: movie length (min)/frame interval (min)/scored area mm^2^ (any other details). Experiment 1: 180 min/1.5 min/0.43428 mm^2^ (shuttles enumerated as the total number in the single imaged area). Experiment 2: 173 min/4.1 min/0.4426 mm^2^ (shuttles enumerated as the total number per field, the central field of 5 fields imaged per well). Experiment 3: 431 min/4.1 min/0.4426 mm^2^ (shuttles enumerated as the total number per field, the central field of 5 fields imaged per well; the 6 Dectin-1^−/−^ wells were randomly selected from 12 imaged wells). “Loaded” means cell contained zymosan–pHrodo particle.

^†^Two-tailed unpaired *t* test. Datasets provided in S1 Data.

**Abbreviations**: BM, bone marrow; PB, peripheral blood.

## Discussion

We previously reported the exchange of cytoplasmic fragments from living neutrophils to macrophages during a wound-stimulated inflammatory response [24], although the physiological purpose of this process was unknown. The data presented here reveal that one purpose of cytoplasmic exchange between neutrophils and macrophages is the transfer of phagocytosed microorganisms. It is readily assumed that when a conidium is found within a particular phagocyte during early infection establishment, then it was that particular phagocyte that first phagocytosed it. We show clearly that is not the case. Conidial shuttling from living neutrophils to macrophages early in fungal infection is an additional and significant aspect of the cell biology of the initial host–pathogen interaction in vivo.

Shuttles could only be identified by careful retrospective analysis of live in vivo imaging files, which presented a substantial challenge to recognizing and studying them and their mechanism. From the 188 independent imaging experiments in this report, we identified 48 stringently defined in vivo conidial shuttles. Using shuttling ascertainment rates as a surrogate for shuttling incidence was sufficient for comparing conditions when exploring shuttling mechanisms. For example, we observed an overall ascertainment rate of 21.4% (30/140 datasets) for biological particles (live or dead fungal spores and zymosan particles), compared to 45.5% (10/22) for β-glucan–coated beads. From a more biological perspective, for in vivo infections following the delivery of 50–100 conidia/inoculum (of which only a minority are initially phagocytosed by neutrophils), the averaged shuttling incidence was at least 19.9% of neutrophil-located spores in the first 3 h of infection. Shuttling was also sufficiently common for multiple occurrences to be recognized in some movies. Collectively, these observations indicate that shuttling is a consistent, recurring phenomenon during infection establishment. Although it comprises only a minority of overall phagocytic events, it still has the potential to impact the outcome of the host–pathogen interaction.

To differentiate shuttling from other mechanisms of pathogen entry into macrophages (such as direct phagocytosis, efferocytosis [9], metaforosis/lateral transfer [15], and trogocytosis [16]), it was critical to observe both the cellular origin of shuttled conidium and the moment of transfer. The only possible way to do this was to perform high-resolution 4-dimensional confocal microscopy with both high spatial and temporal resolution. Our in vivo zebrafish model provided fluorescent labeling clearly distinguishing the two phagocyte lineages, and imaging conditions were optimized for low phototoxicity. While this enabled high spatiotemporal resolution imaging for multiple hours, the imaging volumes often contained considerable biological complexity (high cell densities, cells entering/leaving imaging volume, etc.), which made identifying potential interactions quite challenging. It should also be noted that although the total number of inoculated conidia per experiment was only 50–100 particles, only a fraction were within the imaged volume (Fig 4B and 4C). For these reasons and those mentioned earlier, the shuttling incidence that we report certainly underestimates the absolute rate.

The collective attributes of shuttles distinguish shuttling from all other forms of previously described conidial transfer between leukocytes. In the scenarios we examined, shuttles were unidirectional (neutrophil to macrophage), although we cannot categorically exclude the possibility that shuttling could occur in the opposite direction. Shuttles occurred only in the first hours after inoculation, and very distinctively, donor neutrophils were alive and mobile before and after shuttling and could shuttle one or more conidia. Recipient macrophages were also alive and mobile and could be spore-naïve or preloaded prior to shuttling. Shuttles were preceded by highly regionalized neutrophil–macrophage interactions and occurred through focal cell-to-cell interactions, analogous to an intercellular synapse, that sometimes resulted in tethering of the 2 cells together. Macrophages sometimes received aliquots of neutrophil cytoplasm along with the donated spore. This was demonstrated in some cases to be the concomitant transfer of neutrophil membrane around shuttled conidia, consistent with shuttles being the transfer of conidia-laden phagosomes between donor neutrophil and recipient macrophage rather than just of conidia themselves. This transfer of donor cell membrane also distinguishes shuttling from “nonlytic exocytosis,” as described for the expulsion of previously phagocytosed *C*. *neoformans* from macrophages [13]. Furthermore, although nonlytic exocytosis expels the pathogen from a macrophage, it has not yet been described in the context of a concurrent interaction with another leukocyte lineage. The cytoplasmic exchange did not, however, provide a durable marker of shuttle occurrence because the EGFP signal rapidly disappeared, mostly likely because of acidification of the macrophage phagolysosome, as is dramatically demonstrated by our example of the efferocytosis of an entire bead-laden neutrophil (S3 Fig, S5 Movie).

Emphasizing the distinctive nature of shuttling, our movies captured instances of other forms of pathogen exchange between phagocytes that were clearly not shuttles, including (1) neutrophil-to-neutrophil transfer following complete conidial drop-off and departure by the donor neutrophil (S5A Fig, S7A Movie); (2) neutrophil-to-macrophage transfer following complete conidial drop-off and departure by the donor neutrophil, occurring in proximity to a bona fide shuttle (S5B Fig, S7B Movie); and (3) neutrophil-to-neutrophil transfer via drop-off as in (1), followed by subsequent shuttling of the same conidium from the second neutrophil to a macrophage (S5C Fig, S7C Movie). We did not observe macrophage-to-macrophage lateral transfer of *A*. *fumigatus* conidia as has been previously described [14], likely because this is a later event, predominantly occurring later than 4 h following infection initiation [14].

Both dead and live conidia, labeled with either calcofluor or Alexa Fluor dye, were shuttled. This was consistent with a conidium-directed chemical stimulus driving shuttling and excluded the possibility that shuttling was a conidium-labeling artefact. Furthermore, shuttling of conidia was conserved for two opportunistic fungal pathogen species, but the kinetics of shuttling was pathogen-specific. This suggested that shuttling was driven by a component common to the conidial cell wall of both species, but one present at different levels or exposed to phagocytes to different degrees [33]. Shuttling of zymosan particles provided further evidence locating a shuttling trigger to the cell wall (Fig 5A and 5C), leading β-glucan to be identified as a fungal-derived signal sufficient to drive shuttling of plastic beads (Fig 5B and 5D). A mutant *A*. *fumigatus* strain with reduced β-glucan trended to lower shuttling rates, also consistent with the hypothesis that conidial β-glucan directly drives shuttling. Since zymosan particles were shuttled from murine neutrophils to macrophages in vitro (Fig 7), shuttling seems to be a phenomenon widely conserved among vertebrates and, as for other highly conserved phenomena in host defense, possibly plays an important role in the outcome of infection.

Phagocytosis of fungal pathogens by mammalian leukocytes involves a cluster of pathogen recognition receptors (PRRs), including Dectin-1, Toll-like receptor 2 (TLR2), and Macrophage Mannose Receptor (MMR) [34], and downstream signaling pathways, including spleen tyrosine kinase (Syk) and caspase recruitment domain family member 9 (CARD9) [35]. The main mammalian receptor for β-glucan is Dectin-1, although other receptors are also involved [31,32]. Hence, it was likely that this receptor and the downstream signaling pathways it engages would be involved in conidial shuttling (as well as phagocytosis). A homolog of mammalian Dectin-1 is yet to be identified in the zebrafish genome, but known downstream signaling components such as Syk have been studied [36]. Our in vitro assay showed that for zymosan shuttling between murine cells, there is not an absolute requirement for recipient macrophages to express Dectin-1, but its absence compromises shuttling efficiency. Other shuttle-initiating signals must be involved. Because the neutrophil is clearly viable following the exchange, and because tethering involves only a small portion of the neutrophil membrane, it is improbable that the triggers are broadly displayed “eat me” signals of imminently apoptotic neutrophils such as phosphatidyl serine or calreticulin [37]. However, regionalized display of such signals might be possible. Testing these hypotheses in vivo will be challenging and will require cell-specific and temporally constrained approaches because their global inhibition will inhibit initial neutrophil phagocytosis of conidia, which is a prerequisite for shuttling. Our in vitro systems provide an alternative approach for addressing these mechanistic questions.

**Fig 9:**
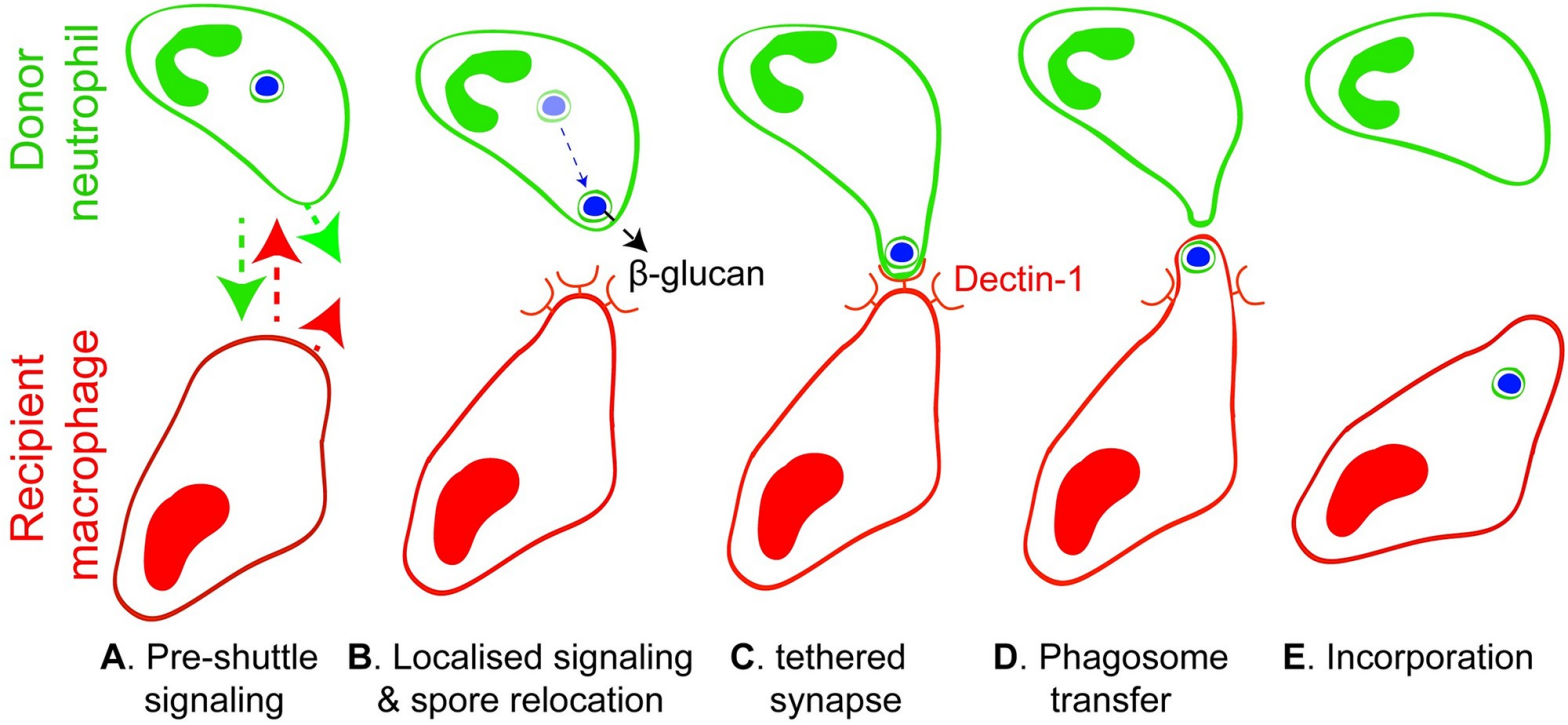
Model of neutrophil-to-macrophage conidial shuttles. The schematic indicates 5 steps in neutrophil-to-macrophage conidial shuttling that accommodate morphological and mechanistic insights from these studies. Undefined signals slow the donor neutrophil and recipient macrophage and bring them into proximity (A), leading to β-glucan–dependent intercellular shuttling signals and spore relocation within the donor cell towards the recipient macrophage (B). An intercellular synapse forms with tethering (C), leading to phagosome transfer (D) and its incorporation into recipient macrophages by a mechanism partially dependent on Dectin-1, at least initially retaining components of the membrane-lined donor cell phagosome (E). Both donor and recipient cells remain active following shuttling, and eventually, both depart (E).

Our current model for shuttling begins with priming of the spore-laden donor neutrophil and its engagement with the recipient macrophage through preshuttle contacts. Within the neutrophil, cytoskeletal rearrangement relocates the conidium within a membrane-lined phagosome towards the side of the neutrophil proximate to the recipient macrophage. The conidium, still within its phagosome, is then transferred from the donor neutrophil to the recipient macrophage. β-glucan–dependent molecular signals are required for shuttling and involve Dectin-1 signaling in the recipient macrophage (Fig 9).

Neutrophil-to-macrophage pathogen shuttling poses other intriguing mechanistic questions. Is it unique to fungal infection or does it occur more widely? Neutrophil-to-macrophage cytoplasm transfer was observed during inflammation [24], suggesting that shuttling may be regulated by inflammatory cytokines. Macrophage cytoplasm transfer to melanoma tumor cells has recently been shown to augment metastatic dissemination and may be another manifestation of this behavior [3]. Is shuttling achieved by repurposing of existing cellular machinery? The tethering of separating participating cells immediately after the interaction points to potential involvement of the neutrophil uropod, a structure under much traction stress and rich in actively rearranging cytoskeletal components such as actin–myosin bundles [38]. Shuttling may be another manifestation of co-opted trogocytosis mechanisms, as described for macrophage-to-macrophage exchange of gram-negative bacteria [16]. However, trogocytosis-associated intercellular bacterial exchanges cannot involve β-glucan signaling.

The most tantalizing question is: what is the impact on the microbiological outcome of the infection? Tied up with this is whether shuttling serves to benefit the host or the pathogen. We recently showed in zebrafish models that macrophages provide an intracellular niche protecting *T*. *marneffei* conidia from neutrophil fungicidal activity [20]. *A*. *fumigatus* conidia are also protected by macrophages from neutrophil fungicidal activities [20,23]. Hence, fungal-driven shuttling may have evolved to optimize the location of invading conidia into the less-hostile intracellular environment of macrophages. Certainly, for these two pathogens, shuttling augments initial conidial redistribution away from the unfavorable neutrophil intracellular environment into their viability-enhancing macrophage intracellular niche. Alternatively, shuttling may be a host defense mechanism aiding adaptive immunity. Neutrophils are ineffective antigen-presenting cells, whereas macrophages specialize in this; therefore, the potential outcome of neutrophil-to-macrophage transfer would be to make pathogen antigens accessible to the adaptive immune system. Delineating the viability outcome for shuttled conidia will require tools for tracing individual shuttled spore fate and/or longitudinal viability throughout the animal (not just in the limited high-magnification imaged volume required to observe its occurrence) and for selectively impairing shuttling but not phagocytosis, neither of which is currently possible in vivo, where shuttling is observed in its most physiological context.

Now that this additional phagocyte behavior during fungal infection establishment has been recognized, its implications must be factored into future understanding of the initial host–pathogen interaction specifically and into the view of neutrophil and macrophage behaviors generally.

## Materials and methods

### Zebrafish

Zebrafish strains were wild type (AB*) carrying single transgenes or combinations of *Tg(mpx*:*EGFP)*^*i114*^ [39]; *Tg(mpeg1*:*Gal4FF)*^*gl25*^ [24]; *Tg(UAS-E1b*:*Eco*.*NfsB-mCherry)*^*c264*^ (Zebrafish International Stock Centre, Eugene, OR, USA); and *Tg(mpeg1*:*mCherry-CaaX)*^*gl26*^ [20]; *Tg(mpx*:*EGFP-CaaX)*^*gl27*^ [20]. Fish were held in the FishCore (Monash University, Melbourne, Australia) aquaria using standard practices. Embryos were held at 28 °C in egg water (0.06 g/L salt [Red Sea, Sydney, Australia]) or E3 medium (5 mM NaCl, 0.17 mM KCl, 0.33 mM CaCl_2_, 0.33 mM MgSO_4_, equilibrated to pH 7.0); at 12 hpf, 0.003% 1-phenyl-2-thiourea (Sigma-Aldrich, St. Louis, MO, USA) was added. All zebrafish embryos and larvae used in experiments were younger than 7 dpf. Zebrafish exhibit juvenile hermaphroditism, so gender balance in embryonic and larval experiments was not a consideration [40].

### Mice

Experiments performed at the Walter and Eliza Hall Institute (Melbourne, Australia) and Boston Children’s Hospital (Boston, MA, USA) used C57BL/6J mice bred in-house. Experiments at University of California San Diego (La Jolla, CA, USA) used B6.129S6-Clec7a^tm1Gdb^/J [41] and C57BL/6J mice sourced directly from Jackson Laboratories (Bar Harbor, ME, USA) and temporarily housed locally.

### Ethics and biosafety statement

All animal experiments followed appropriate NHMRC guidelines. Zebrafish experiments were conducted under protocols approved by Ethics Committees of Monash University (MAS/2010/18, MARP/2015/094). Zebrafish experiments were performed under Institution Biosafety Committee Notifiable Low Risk Dealing (NLRD) approval PC2-N23-10 (Monash University). *T*. *marneffei* and *A*. *fumigatus* were assigned to risk group 2 at the time these approvals were granted. In most jurisdictions, including endemic regions, *T*. *marneffei* is a risk group 2 organism. Protocols for mouse experiments were approved by the Animal Ethics Committee of the Walter and Eliza Hall Institute (2010.007) or the Institutional Animal Care and Use Committees of Boston Children’s Hospital (16-07-3223) or University of California San Diego (S18236).

### *T*. *marneffei* and *A*. *fumigatus*

The *T*. *marneffei* strain SPM4 used in this study is a derivative of the FRR2161-type strain [42]. For *A*. *fumigatus*, wild-type *CEA10* [43] and mutant Δ*gel1*Δ*gel7*Δ*cwh41* [28] triple-mutant strains were used. Throughout this report, the terms “spore” and “conidium” both refer to asexual fungal spores.

To prepare fresh conidia for injection, *T*. *marneffei* and *A*. *fumigatus* conidial suspensions were inoculated onto solidified ANM medium and cultured at 25 °C for 10–12 days when the cultures were conidiating. Conidia were washed from the plate with 0.001% Tween 80 solution, filtered, sedimented (6,000 rpm, 10 min), resuspended in 0.001% Tween 80 solution, and stored at 4 °C. For inoculation, conidia were resedimented and resuspended in Phosphate-Buffered Saline (PBS). Fungal colony-forming unit (CFU) numbers per embryo were determined as previously described [20].

Cold inactivation of *T*. *marneffei* conidia and calcofluor staining was as described previously [20,24]. To inactivate *A*. *fumigatus* conidia, they were γ-irradiated with 10 kGy [44] from a Gammacell 40 Exactor (Best Theratronics, Ottawa, Ontario, Canada) as previously described [20] and verified as dead by lack of growth after 5 days incubation. Irradiated conidia still stained well with calcofluor and were microinjected at the same dilution of stock as used for live conidia.

### Zebrafish infection with *T*. *marneffei* and *A*. *fumigatus*

Freshly prepared *T*. *marneffei* and *A*. *fumigatus* conidia stocks for these experiments were stored at 4 °C for <2 months. For inoculation, 52 hpf tricaine-anesthetized embryos were mounted on an agar mold with head/yolk within the well and tail laid flat on the agar. The fungal conidial suspension was inoculated intramuscularly into a somite aligned to the yolk extension tip for local infection [24,45] using a standard microinjection apparatus (Pico-Injector Microinjection System; Harvard Apparatus, Holliston, MA, USA) and thin-wall filament borosilicate glass capillary microinjection needle (SDR Clinical Technology, prepared using a P-2000 micropipette puller; Sutter Instruments, Novato, CA, USA). Inoculated embryos were held at 28 °C. The delivered conidial dosage was verified by immediate CFU enumeration on a group of injected embryos [20]. It took approximately 10 min to commence imaging after inoculation; in this report, the zero time point (*t* = 0) is taken as the beginning of imaging.

### Calcofluor and Alexa Fluor 405 staining of conidia

For calcofluor staining, spores were incubated in 10 mM calcofluor White (Sigma-Aldrich) for 30 min, followed by 2 washing steps and resuspension in distilled water.

To stain fungal conidia with Alexa Fluor 405 NHS Succinimidyl Ester (Life Technologies, Carlsbad, CA, USA), 10 μL of Alexa Fluor dye was added to 200 μL of suspended conidia with gentle shaking at room temperature for 30 min, followed by washing steps with PBS (pH 8) and 25 mM Tris (pH 8.5), and finally resuspended in PBS (pH 7), according to the supplier’s protocol.

### β-glucan immunofluorescence microscopy

Detection of β-glucan exposure on nongerminated *T*. *marneffei* (FRR2161) and *A*. *fumigatus* (CEA10) dormant conidia was by immunofluorescence microscopy using anti-β-1,3 glucan linkage primary antibody (mouse IgG kappa; Biosupplies, Bundoora, Australia) at 1:500 dilution and a goat anti-mouse IgG (Alexa Fluor 488; Abcam, Cambridge, UK) as secondary antibody at 1:750 dilution. Fungal particles were poststained with calcofluor. *S*. *cerevisiae* cell-wall ghosts were used as a positive control [46], and no primary antibody as negative controls. Fluorescence was detected on a Nikon C2 confocal microscope (Nikon, Tokyo, Japan) and images processed in Imaris to generate maximum intensity projections with orthogonal views and 3-dimensional-surface–rendered images.

### Zymosan particles

Zymosan A particles from *S*. *cerevisiae* (Sigma-Aldrich) with an average size of 3 μm were stained by calcofluor as for fungal conidia prior to microinjection.

### Plastic beads

SPHERO fluorescent light yellow particles, high-intensity–sized 1.7–2.2 μm (SPHEROTECH, Lake Forest, IL, USA) (concentration 1.0% w/v in deionized water with 0.02% sodium azide), were used. These particles were kept at room temperature. Excitation and emission wavelengths were 400 and 450 nm, respectively. Customized commercially prepared light yellow particles coated with laminarin as a source of β-glucan (SPHERO Laminarin Polysaccharide Fluorescent Particles, Light Yellow, 1.5–1.99 μm, Catalog no. LPFP1545-2, Lot no. AH01; SPHEROTECH) were also used. Laminarin for coating was from *Laminaria digitata* (primarily poly(β-Glc-[1→3]) with some β-[1→6] interstrand linkages and branch points; Sigma-Aldrich) [32,47].

### In vitro studies using murine phagocytes

For the experiments of Fig 7, primary C57BL/6J mouse bone marrow leukocytes were collected and purified as previously described [48,49]. Macrophages were plated at 5 × 10^3^ in an 8-well plate and incubated in Dulbecco’s modified Eagle’s medium with 10% fetal bovine serum and 20% L-929 conditioned medium for 16 h. Primary bone marrow neutrophils were preloaded with Alexa-Fluor-488–labeled opsonized zymosan particles for 1 h at 37 °C in Dulbecco’s modified Eagle’s medium and 10% fetal bovine serum. Preloaded neutrophils were added to adherent macrophages at 10^5^ cells per well. Imaging was performed on a Nikon Biostation IM-Q at 37 °C/10% CO_2_.

The experiments of Fig 8 and S2 Table were conducted in 96-well plates. Either bone marrow or peripheral blood neutrophils, pooled from 8 donor mice, were used (5 × 10^4^/well and 3 × 10^5^/well, respectively). Neutrophils were labeled with CellTracker Green dye (200 nM, 15 min, 37 °C; Thermo Fisher Scientific, Waltham, MA, USA), primed with G-CSF (100 ng/mL for 1 h to maintain viability), preloaded with pHrodo Red Zymosan Bioparticles (1 h incubation at 1:1 particle/cell ratio), and then overlaid on adherent bone-marrow–derived macrophages (10^4^/well) labeled with CellTracker Deep Red dye (1 μM, 15 min, 37 °C; Thermo Fisher Scientific). Imaging used an ImageXpress Micro confocal microscope (Molecular Devices, San Jose, CA, USA) operating MetaXpress (Version 6.2.5) acquisition software. Three nonoverlapping 1.4 × 1.4 mm zones within each well were imaged for red, green, and far-red fluorescence (excitation/emission at 531/593, 475/536, and 634/692 nm, respectively) at 3-min intervals for up to 306 min. Frame sequences were constructed in Fiji and viewed in Imaris for scoring. To derive the descriptive statistics, cell types (neutrophils and macrophages, loaded and unloaded) were manually counted, aided by a 4 × 4 grid dividing this region into 16 squares, each square having an area of 0.1225 mm^2^. “Loaded” phagocytes were defined as having at least one pHrodo-positive zymosan particle.

Experiments of Table 1 using Dectin-1^−/−^ bone-marrow–derived macrophages used similar conditions, except that the neutrophil concentrations were as follows: Experiment 1, bone marrow neutrophils 5 × 10^4^/well and peripheral blood neutrophils 10^5^/well; Experiment 2, bone marrow neutrophils 2 × 10^5^/well; Experiment 3, bone marrow neutrophils, 1.5 × 10^5^/well. Imaging used the following: Experiment 1, Ultraview Vox Spinning Disk Confocal microscope (Perkin Elmer, Waltham, MA, USA) using a Hamamatsu EMCCD 14-bit 1,000 × 1,000 camera (Hamamatsu City, Japan), collecting 3 fluorescence channels at 1.5-min intervals using Volocity (Quorum Technologies, Lewes, UK) software; Experiments 2 and 3, Leica DMi8 Thunder microscope (Leica, Wetzlar, Germany) running LAS X software (version 3.6).

### Microscopy and image processing

Routine bright-field and fluorescence imaging of zebrafish used an Olympus MVX10 stereo dissecting microscope (Olympus, Tokyo, Japan) with MV PLAPO 1× and 2×C objectives, fitted with an Olympus DP72 camera and Cellsense standard software, version 1.11.

Confocal intravital microscopy used a Zeiss LSM 5 Live with a Plan-Apochromat 20×, 0.8 NA objective (Zeiss, Oberkochen, Germany). ZEN software (2012, black edition, 64-bit) was used for acquisition, and images were 16-bit 512 × 512 pixels. Z-depth ranged from 35–130 μm (72 ± 23 μm) and was composed of 20–40 slices (31 ± 4). Time intervals between z-stacks were set as zero to perform continuous acquisition (z-stack acquisition took 33.24 ± 9.50 s). Excitatory laser wavelengths were 405 nm for calcofluor, 489 nm for EGFP, and 561 nm for mCherry. Emission detection used a BP495-555 filter for calcofluor and EGFP and an LP575 filter for mCherry. Excitation/emission conditions for light yellow particles were the same as for calcofluor.

Details of microscopes, cameras, and acquisition software used for other experiments are provided with their respective methods.

### Image processing and analysis

All fluorescent image analyses were performed primarily in Imaris (BitPlane) software version 8.1.2 on Venom (Intel Core i7-4770 Processor, 3.4 GHz) or Titan (Intel Xeon Processor E5-2680 v2 [2 × 2.80 GHz], 128 GB RAM) computers (Monash Micro Imaging facility, Monash University). Some analyses used Fiji (ImageJ 1.46r) and MATLAB (The MathWorks). For Fig 4A, data were analyzed in the R program using ggplot2 as previously [25,50]. Figures were constructed using Adobe Illustrator CS5 (version 15.0.0).

### Shuttle detection and definition

All in vivo shuttles were detected by systematic manual frame-by-frame inspection of movies. For these studies, a “shuttle” was stringently defined as a spore transfer event meeting all of the following criteria: (1) both donor and recipient cells were imaged in toto before, during, and after the shuttle; (2) both donor and recipient cells demonstrated their viability before and after shuttling by migration; (3) the moment of donor-to-recipient cell transfer was visualized; and (4) z-stack viewing unequivocally confirmed that conidia were within donor and recipient cells prior to and after the shuttle. Experience taught that shuttles were most easily recognized by watching movies in reverse and tracing the source of individual macrophage-located conidia. The identity of all 46 unequivocal in vivo shuttles meeting these criteria contributing to this report is assigned in S1 Fig and S1 Table and is indicated throughout the report. When referring to “unselected” series/examples/imaging sequences, we mean the full dataset of S1 Fig and S1 Table.

### ShuttleFinder software

The confocal time series were imported into MATLAB (version 8.1.0.604, R2013a) utilizing the bioformats toolbox [51], and the conidia were tracked with PhagoSight [26]. The data input consisted of confocal time series with three channels, each containing the fluorescence of one cell type. PhagoSight was designed to track phagocytes in confocal time series. Since conidia are smaller than phagocytes, the reduction step of PhagoSight was only applied to large files that would have taken more than 3 days to process without it. To reduce the likelihood of false negatives, the automatic determined threshold for background separation by PhagoSight was lowered by 10%. For each file, only the longest tracks were analyzed (upper third of track length over time). PhagoSight was used in command line mode without user interaction to allow for automated processing, using the MASSIVE cluster [52].

PhagoSight calculates a bounding box, which described the volume surrounding each tracked spore for each time frame. The intensity of the voxels in the two channels describing neutrophils and macrophages was summed over this bounding box, and a proportional index r between both was calculated:

$$\frac{\sum_{xyz}I_{\text{red}}(x,y,z)-\sum_{xyz}I_{\text{green}}(x,y,z)}{\sum_{xyz}I_{\text{red}}(x,y,z)+\sum_{xyz}I_{\text{green}}(x,y,z)},$$

with *I* describing the intensity of one channel. This ratio was smoothed with a moving average filter over three imaging frames to remove noise caused by imperfections in the tracking process. Subsequently, a point in a track was defined as being in a macrophage (red channel) if the values lay between 1 and 0.2, conversely in a neutrophil (green channel) for a value between −0.2 and −1. To be classified as a candidate shuttle event, the r-values for a conidium track had to pass from either −0.2 to 0.2 (for a neutrophil-to-macrophage shuttle) or vice versa.

The time the tracked conidium reached the threshold was considered the beginning of the shuttle. The end of the shuttle event was defined by the track leaving the threshold area.

### Statistics

Descriptive and analytical statistics were prepared in Prism 5.0c (GraphPad Software Inc., San Diego, CA, USA). Unless otherwise stated, data are mean ± SD, with *p*-values generated from two-tailed unpaired *t* tests for normally distributed continuous variables and chi-squared tests for categorical variables.

### Datasets

All numerical datasets are provided in S1 Data (main figures and table) and S2 Data (supplementary files). S1 Table lists the imaging file datasets and correlates the still images in all figures with their respective source movie files.

## Supporting information

S1 Fig Imaging datasets. Details of the imaging datasets in which the defining set of shuttles of 13 *T*. *marneffei* (A) and 7 *A*. *fumigatus* (B) conidia meeting stringent definition criteria were found. Graphs show the distribution of imaging file lengths, which files contained a shuttle (black columns), the shuttle ID (#), the shuttle movie length (L), and the time of shuttle (yellow mark in black column and red numeral in min). The two distributions of movie lengths are not significantly different (*p* = 0.1985, Mann–Whitney U test). Corresponds to Figs 1, 2 and 4 and S1 Table. Datasets are provided in S2 Data.
(TIF)

S2 Fig Examples of probable shuttles. A variety of shuttles of conidia or particles (blue) from *Tg(mpx*:*EGFP)* neutrophils (green) to *Tg(mpeg1*:*Gal4FF)×(UAS-E1b*:*Eco*.*NfsB-mCherry)* macrophages (red). In each example, panels include isometric orthogonal *yz* and *xz* views corresponding to the *xy* maximal intensity projection and indicate the time in min from start of movie. Colored arrowheads indicate the conidium/particle within donor neutrophil (green), at the point of intercellular transfer (white) and in the recipient macrophage (red). (A–C) Probable shuttles of conidia. (A) A probable shuttle in which the conidium is not clearly resolved as fully contained within the donor neutrophil. (B–C) Probable shuttles of conidia in which the point of cell-to-cell contact is not clearly displayed. (D) An example of a crowded field with multiple neutrophils and macrophages in which initially there are neutrophils laden with conidia and by the end conidia are mostly within macrophages, although the transfer of conidia is not clearly seen. Scales as shown. Stills in A–D correspond to S4A–S4D Movie, respectively. *Eco*.*Nfsb*, *E*. *coli* nitroreductase; EGFP, enhanced green fluorescent protein; *Gal4FF*, engineered form of *S*. *cerevisiae* Gal4 transcriptional activator; *mpeg1*, macrophage-expressed gene 1; *mpx*, myeloid-specific peroxidase; *Tg*, transgenic; *UAS-E1b*, upstream activating sequence fused to minimal adenovirus E1b promoter.
(TIF)

S3 Fig Efferocytosis of an entire bead-laden neutrophil. Phagocytosis of inert 2-μm plastic beads (blue) by *Tg(mpx*:*EGFP)* neutrophils (green), followed by efferocytosis of the whole particle-laden neutrophil by a *Tg(mpeg1*:*Gal4FF)×(UAS-E1b*:*Eco*.*NfsB-mCherry)* macrophage (red). Subsequently, the EGFP signal of the engulfed neutrophil is extinguished although the Alexa Fluor signal (blue) of the plastic beads persists (right panel). Panels include isometric orthogonal *yz* and *xz* views corresponding to the *xy* maximal intensity projection and indicate the time in min from start of movie. White arrowheads follow the neutrophil of interest through the process. Scale as shown. Stills from S5B Movie. *Eco*.*Nfsb*, *E*. *coli* nitroreductase; EGFP, enhanced green fluorescent protein; *Gal4FF*, engineered form of *S*. *cerevisiae* Gal4 transcriptional activator; *mpeg1*, macrophage-expressed gene 1; *mpx*, myeloid-specific peroxidase; *Tg*, transgenic; *UAS-E1b*, upstream activating sequence fused to minimal adenovirus E1b promoter.
(TIF)

S4 Fig β-glucan exposure on the surface of conidial inoculates. Immunofluorescence detection of 1,3 β-glucan (green) on the surface of *T*. *marneffei* and *A*. *fumigatus* conidia prepared as inoculates (counterstained with calcofluor, blue), displayed as maximum intensity projections (left) and surface-rendered views (right). Negative controls omitted primary antibody. *S*. *cerevisiae* ghosts serve as a positive technical control for β-glucan detection.
(TIF)

S5 Fig Conidial transfer between phagocytes by processes other than shuttling. These examples are from experiments using Δ*gel1*Δ*gel7*Δ*cwh41 A*. *fumigatus* conidia. (A) Neutrophil-to-neutrophil transfer involving conidial drop-off and departure by the donor neutrophil and reuptake of the deposited conidium by a second neutrophil, two examples in the same field of view. (B) Neutrophil-to-macrophage transfer involving conidial drop-off and departure by the donor neutrophil and reuptake of the deposited conidium by a macrophage, coincidentally occurring in proximity to a bona fide shuttle occurring slightly later. (C) Neutrophil-to-neutrophil transfer via drop-off as in (A), followed by subsequent shuttling of the same conidium from the second neutrophil to a macrophage. Arrowheads indicate the conidium of interest (numbered 1,2 where necessary) within the neutrophil (green), during the extracellular drop-off period (blue), at the time of shuttling (white), and within the macrophage (red). Scales as shown. Stills from S7A–S7C Movie. *cwh41*, *A*. *fumigatus* α-glucosidase 1; *gel*, *A*. *fumigatus* β-1,3-glucanosyltransferase.
(TIF)

S1 Movie Six examples of live *T*. *marneffei* conidial shuttles. Shuttles are of live calcofluor-stained conidia (blue) from a *Tg(mpx*:*EGFP)* neutrophil (green) to a *Tg(mpeg1*:*Gal4FF)×(UAS-E1b*:*Eco*.*NfsB-mCherry)* macrophage (red). Movies run in series. Movies are paused at the moment of shuttling, with the point of transfer labeled (white arrow). (A) Standard shuttle (corresponds to Fig 1A). (B) Standard shuttle with tethered recipient macrophage (corresponds to Fig 1E). (C) Standard shuttle with tethered donor neutrophil (corresponds to Fig 2A). (D) Standard shuttle with tethered departing donor neutrophil. (E) Standard shuttle of multiple spores in quick succession (corresponds to Fig 2B). (F) Two conidia shuttled asynchronously (corresponds to Fig 2C). *Eco*.*Nfsb*, *E*. *coli* nitroreductase; EGFP, enhanced green fluorescent protein; *Gal4FF*, engineered form of *S*. *cerevisiae* Gal4 transcriptional activator; *mpeg1*, macrophage-expressed gene 1; *mpx*, myeloid-specific peroxidase; *Tg*, transgenic; *UAS-E1b*, upstream activating sequence fused to minimal adenovirus E1b promoter.
(MP4)

S2 Movie Six examples of *A*. *fumigatus* conidial shuttles. Shuttles are of live calcofluor-stained conidia (blue) from a *Tg(mpx*:*EGFP)* neutrophil (green) to a *Tg(mpeg1*:*Gal4FF)×(UAS-E1b*:*Eco*.*NfsB-mCherry)* macrophage (red). Movies run in series. Movies are paused at the moment of shuttling, with the point of transfer labeled (white arrow). (A) Standard shuttle (corresponds to Fig 2D). (B) Standard shuttle. (C) Standard shuttle of Alexa-Fluor-405–stained conidium (corresponds to Fig 2E). (D) Shuttling involving a highly polarized and tethered neutrophil and macrophage interaction. (E) Two independent shuttles occurring in the same field. (corresponds to Fig 2F). (F) Two conidia shuttled together. *Eco*.*Nfsb*, *Escherichia coli* nitroreductase; EGFP, enhanced green fluorescent protein; *Gal4FF*, engineered form of *S*. *cerevisiae* Gal4 transcriptional activator; *mpeg1*, macrophage-expressed gene 1; *mpx*, myeloid-specific peroxidase; *Tg*, transgenic; *UAS-E1b*, upstream activating sequence fused to minimal adenovirus E1b promoter.
(MP4)

S3 Movie Four examples of dead *T*. *marneffei* conidial shuttles. Shuttles are of dead calcofluor-stained conidia (blue) from a *Tg(mpx*:*EGFP-CaaX)* neutrophil (green) to a *Tg(mpeg1*:*mCherry-CaaX)* macrophage (red). Movies run in series. Movies are paused at the moment of shuttling, with the point of transfer labeled (white arrow). These reporter lines localize the fluorophore to the membrane, enabling these movies to display volume-rendered version of donor neutrophil and recipient macrophages in parallel (right panels). (A–C) Three independent shuttles of individual dead conidia, all occurring in the same movie (shuttle (A) corresponds to Fig 3A). (D) Standard shuttle of a dead conidium. EGFP, enhanced green fluorescent protein; *mpeg1*, macrophage-expressed gene 1; *mpx*, myeloid-specific peroxidase; *Tg*, transgenic.
(MP4)

S4 Movie Four examples of probable conidial shuttles not meeting all definition criteria. Shuttles are of live calcofluor-stained conidia (blue) from a *Tg(mpx*:*EGFP)* neutrophil (green) to a *Tg(mpeg1*:*Gal4FF)×(UAS-E1b*:*Eco*.*NfsB-mCherry)* macrophage (red). Movies run in series. Movies are paused at the moment of shuttling, with the point of transfer labeled (white arrow). (A) Probably shuttle in which the conidium is not unequivocally resolved as being within the donor neutrophil (corresponds with S2A Fig). (B,C) Probable shuttles in which direct intercellular contact between donor neutrophil and recipient macrophage is not clearly displayed (corresponds with S2B and S2C Fig). (D) Crowded field in which there are initially neutrophil-laden conidia and, at the end, macrophage-laden conidia, but the crowding obscures probable conidial shuttling (corresponds with S2D Fig). *Eco*.*Nfsb*, *E*. *coli* nitroreductase; EGFP, enhanced green fluorescent protein; *Gal4FF*, engineered form of *S*. *cerevisiae* Gal4 transcriptional activator; *mpeg1*, macrophage-expressed gene 1; *mpx*, myeloid-specific peroxidase; *Tg*, transgenic; *UAS-E1b*, upstream activating sequence fused to minimal adenovirus E1b promoter.
(MP4)

S5 Movie Examples of shuttles of nonconidial particles. Shuttles are nonconidial particles (blue) from a *Tg(mpx*:*EGFP)* neutrophil (green) to a *Tg(mpeg1*:*Gal4FF)x(UAS-E1b*:*Eco*.*NfsB-mCherry)* macrophage (red). Movies run in series. Movies are paused at the moment of shuttling, with the point of transfer labeled (white arrow). (A) Shuttle of zymosan particle (corresponds to Fig 6C). (B) Efferocytosis (not a shuttle) of whole neutrophil laden with plastic beads (corresponds to S3 Fig). (C) Shuttle of β-glucan–coated plastic beads (corresponds to Fig 6D). *Eco*.*Nfsb*, *E*. *coli* nitroreductase; EGFP, enhanced green fluorescent protein; *Gal4FF*, engineered form of *S*, *cerevisiae* Gal4 transcriptional activator; *mpeg1*, macrophage-expressed gene 1; *mpx*, myeloid-specific peroxidase; *Tg*, transgenic; *UAS-E1b*, upstream activating sequence fused to minimal adenovirus E1b promoter.
(MP4)

S6 Movie Examples of zymosan shuttles between murine neutrophils and macrophages in vitro. Two shuttles of zymosan particles between murine neutrophils preloaded with Alexa-Fluor-488–labeled zymosan and adherent murine macrophages in an in vitro assay. Photomicrographs are bright-field views overlaid with green fluorescence channel. White arrows in paused frames indicate the shuttle. Time stamps are provided in the corresponding Fig 7 stills. (A) Shuttle (arrowed) (corresponds with Fig 7A). (B) Shuttle (arrowed) (corresponds with Fig 7B). (C–E) Three examples of zymosan–pHrodo shuttles corresponding to Fig 8A–8C. Neutrophils, green; Macrophages, red; zymosan–pHrodo false-colored blue. (F) Shuttle of zymosan–pHrodo from a wild-type neutrophil to a Dectin1^−/−^ macrophage (corresponds with Fig 8F).
(MP4)

S7 Movie Examples of conidial transfer between phagocytes by processes other than shuttling. Examples are from experiments using Δ*gel1*Δ*gel7*Δ*cwh41 A*. *fumigatus* conidia. (A) Neutrophil-to-neutrophil transfer with complete conidial drop-off and departure by the donor neutrophil, two examples in the same field of view (corresponds with S5A Fig). (B) Neutrophil-to-macrophage transfer with complete conidial drop-off and departure by the donor neutrophil, occurring in proximity to a bona fide shuttle occurring slightly later (corresponds with S5B Fig). (C) Neutrophil-to-neutrophil transfer via drop-off as in (A), followed by subsequent shuttling of the same conidium from the second neutrophil to a macrophage (corresponds with S5C Fig). White arrows indicate conidia of interest at key points in the transfer process. *cwh*, *A*. *fumigatus* α-glucosidase 1; *gel*, *A*. *fumigatus* β-1,3-glucanosyltransferase.
(MP4)

S1 Table List of shuttles. (DOCX)

S2 Table Shuttling incidence for in vitro assays with zymosan–pHrodo and wild-type murine neutrophils and macrophages. (DOCX)

S1 Data Numerical data related to main text figures and table. (XLSM)

S2 Data Numerical data related to supplementary files. (XLSX)

## Abbreviations

CARD9: caspase recruitment domain family member 9
CFU: colony-forming unit
*cwh*: *Aspergillus*. *fumigatus* α-glucosidase I
dpf: days postfertilization
*Eco*.*Nfsb*: *Escherichia coli* nitroreductase
EGFP: enhanced green fluorescent protein
*Gal4FF*: engineered form of *Saccharomyces cerevisiae* Gal4 transcriptional activator
*gel*: *A*. *fumigatus* β-1,3-glucanosyltransferase
MMR: macrophage mannose receptor; *mpeg1*, macrophage-expressed gene 1
*mpx*: myeloid-specific peroxidase
NLRD: notifiable low risk dealing
PBS: phosphate-buffered saline
PRR: pathogen recognition receptor
Syk: spleen tyrosine kinase
*Tg*: transgenic
TLR2: toll-like receptor 2
*UAS-E1b*: upstream activating sequence fused to minimal adenovirus E1b promoter
