# Supplementary figures and images for: β-glucan–dependent shuttling of conidia from neutrophils to macrophages occurs during fungal infection establishment
Source: PLoS Biol. 2019 Sep 4;17(9):e3000113. doi: 10.1371/journal.pbio.3000113 (PMC6746390; doi:10.1371/journal.pbio.3000113)

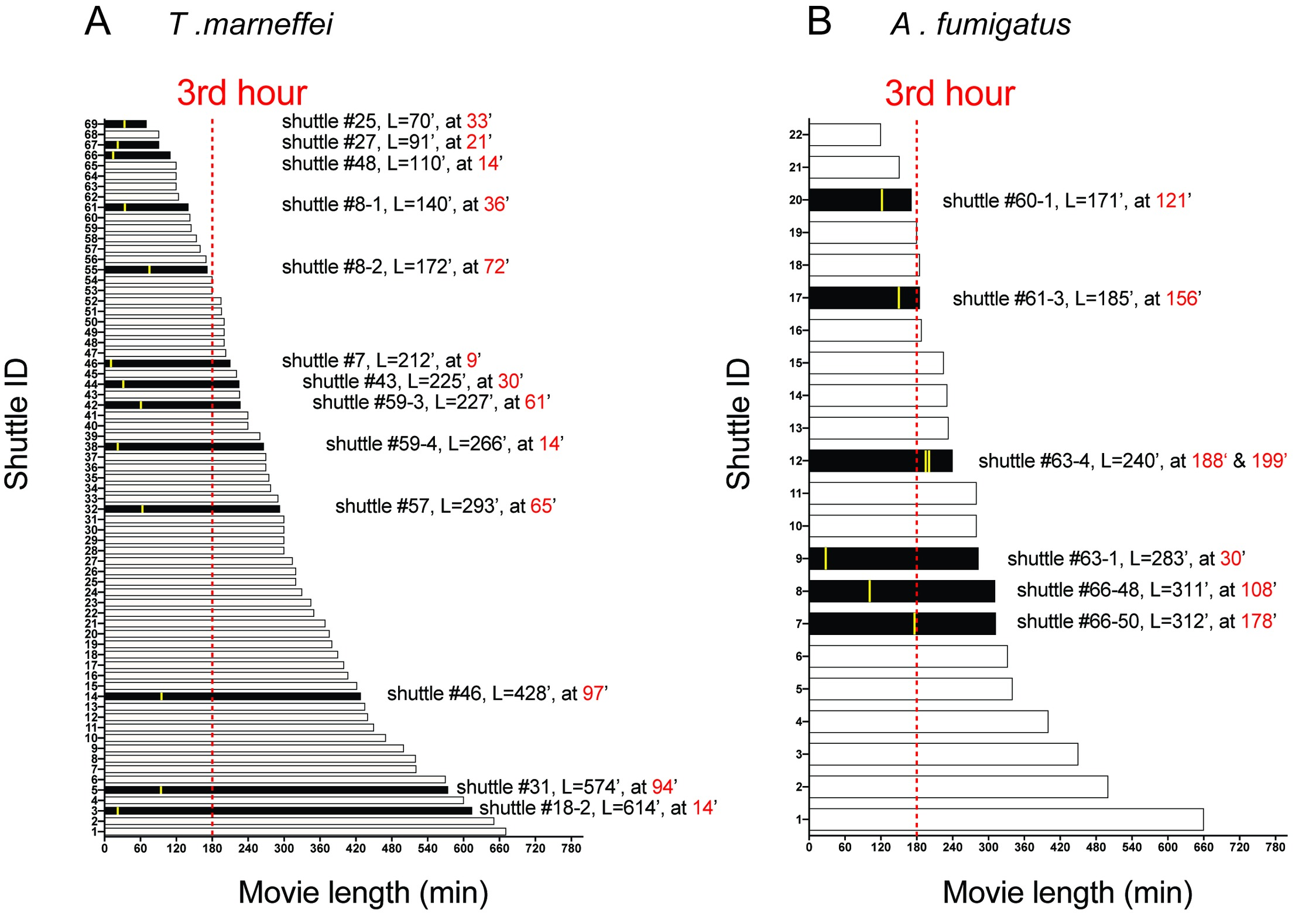

Supplement: S1 Fig — Details of the imaging datasets in which the defining set of shuttles of 13 T. marneffei (A) and 7 A. fumigatus (B) conidia meeting stringent definition criteria were found. Graphs show the distribution of imaging file lengths, which files contained a shuttle (black columns), the shuttle ID (#), the shuttle movie length (L), and the time of shuttle (yellow mark in black column and red numeral in min). The two distributions of movie lengths are not significantly different (p = 0.1985, Mann–Whitney U test). Corresponds to Figs 1, 2 and 4 and S1 Table. Datasets are provided in S2 Data. (TIF) [file pbio.3000113.s001.tif]

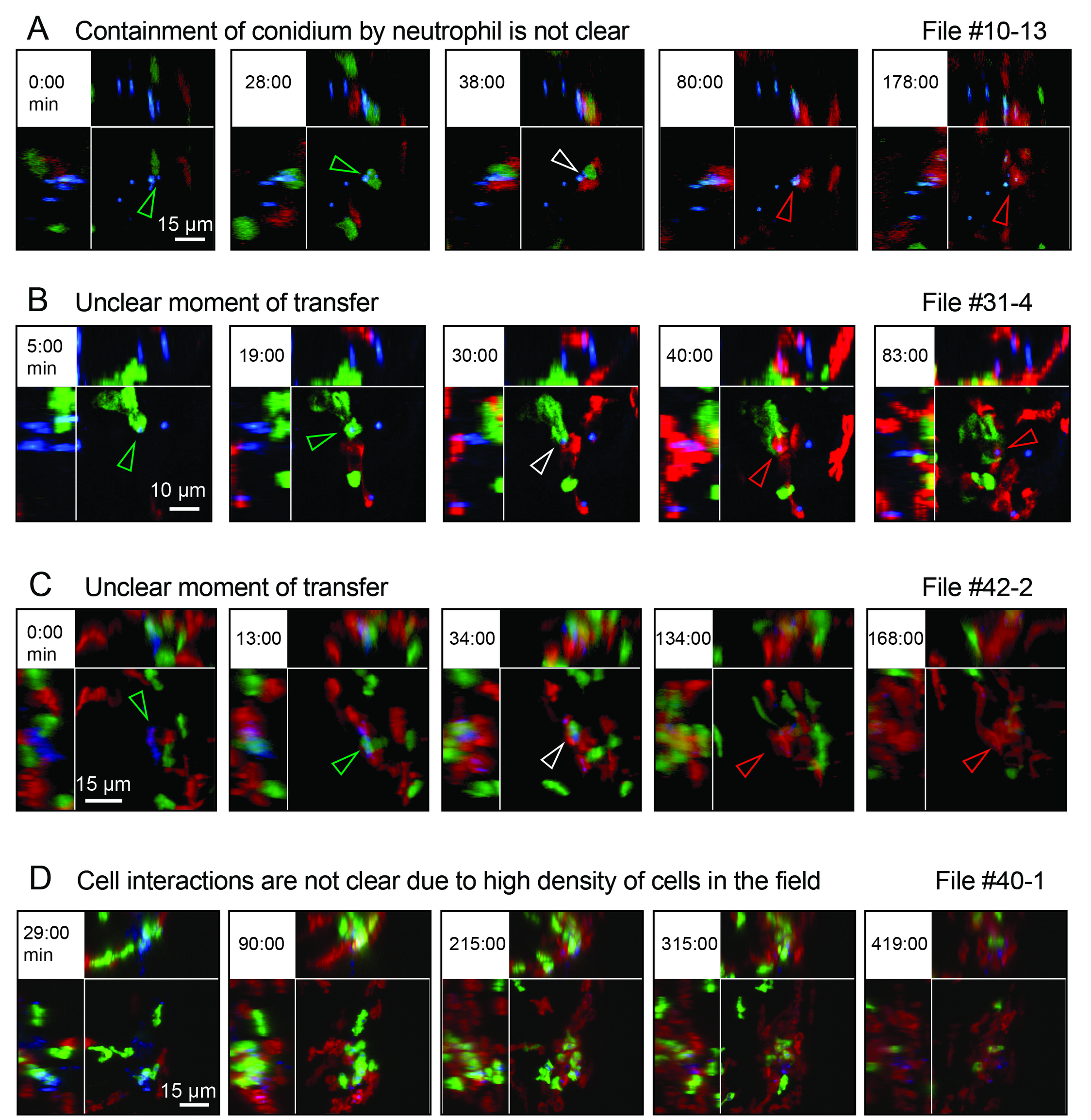

Supplement: S2 Fig — A variety of shuttles of conidia or particles (blue) from Tg(mpx:EGFP) neutrophils (green) to Tg(mpeg1:Gal4FF)×(UAS-E1b:Eco.NfsB-mCherry) macrophages (red). In each example, panels include isometric orthogonal yz and xz views corresponding to the xy maximal intensity projection and indicate the time in min from start of movie. Colored arrowheads indicate the conidium/particle within donor neutrophil (green), at the point of intercellular transfer (white) and in the recipient macrophage (red). (A–C) Probable shuttles of conidia. (A) A probable shuttle in which the conidium is not clearly resolved as fully contained within the donor neutrophil. (B–C) Probable shuttles of conidia in which the point of cell-to-cell contact is not clearly displayed. (D) An example of a crowded field with multiple neutrophils and macrophages in which initially there are neutrophils laden with conidia and by the end conidia are mostly within macrophages, although the transfer of conidia is not clearly seen. Scales as shown. Stills in A–D correspond to S4A–S4D Movie, respectively. Eco.Nfsb, E. coli nitroreductase; EGFP, enhanced green fluorescent protein; Gal4FF, engineered form of S. cerevisiae Gal4 transcriptional activator; mpeg1, macrophage-expressed gene 1; mpx, myeloid-specific peroxidase; Tg, transgenic; UAS-E1b, upstream activating sequence fused to minimal adenovirus E1b promoter. (TIF) [file pbio.3000113.s002.tif]

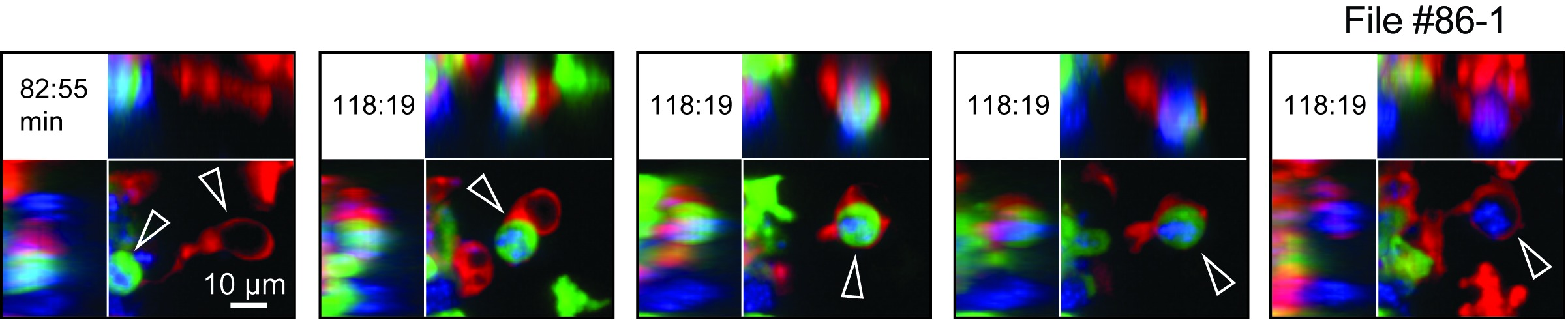

Supplement: S3 Fig — Phagocytosis of inert 2-μm plastic beads (blue) by Tg(mpx:EGFP) neutrophils (green), followed by efferocytosis of the whole particle-laden neutrophil by a Tg(mpeg1:Gal4FF)×(UAS-E1b:Eco.NfsB-mCherry) macrophage (red). Subsequently, the EGFP signal of the engulfed neutrophil is extinguished although the Alexa Fluor signal (blue) of the plastic beads persists (right panel). Panels include isometric orthogonal yz and xz views corresponding to the xy maximal intensity projection and indicate the time in min from start of movie. White arrowheads follow the neutrophil of interest through the process. Scale as shown. Stills from S5B Movie. Eco.Nfsb, E. coli nitroreductase; EGFP, enhanced green fluorescent protein; Gal4FF, engineered form of S. cerevisiae Gal4 transcriptional activator; mpeg1, macrophage-expressed gene 1; mpx, myeloid-specific peroxidase; Tg, transgenic; UAS-E1b, upstream activating sequence fused to minimal adenovirus E1b promoter. (TIF) [file pbio.3000113.s003.tif]

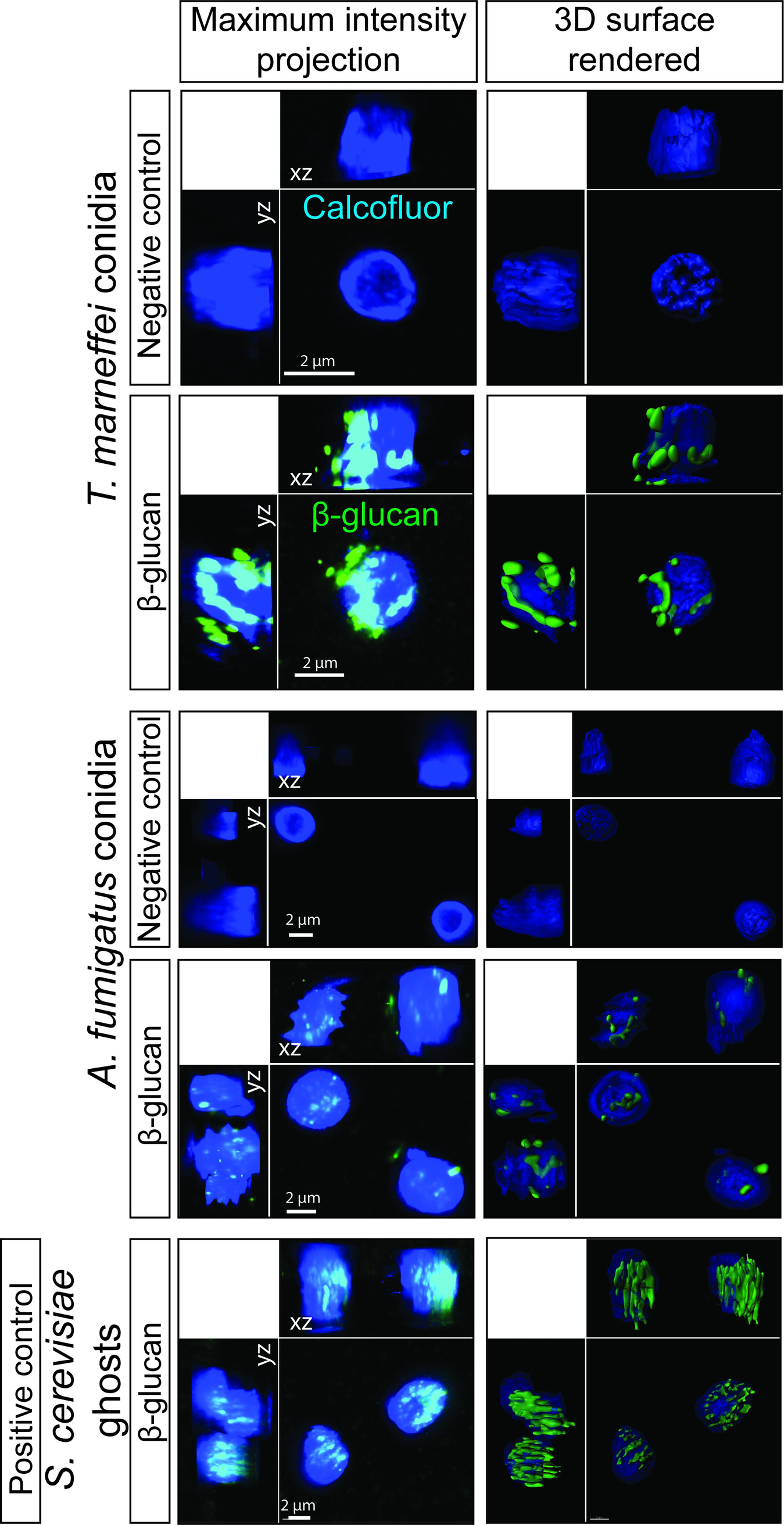

Supplement: S4 Fig — Immunofluorescence detection of 1,3 β-glucan (green) on the surface of T. marneffei and A. fumigatus conidia prepared as inoculates (counterstained with calcofluor, blue), displayed as maximum intensity projections (left) and surface-rendered views (right). Negative controls omitted primary antibody. S. cerevisiae ghosts serve as a positive technical control for β-glucan detection. (TIF) [file pbio.3000113.s004.tif]

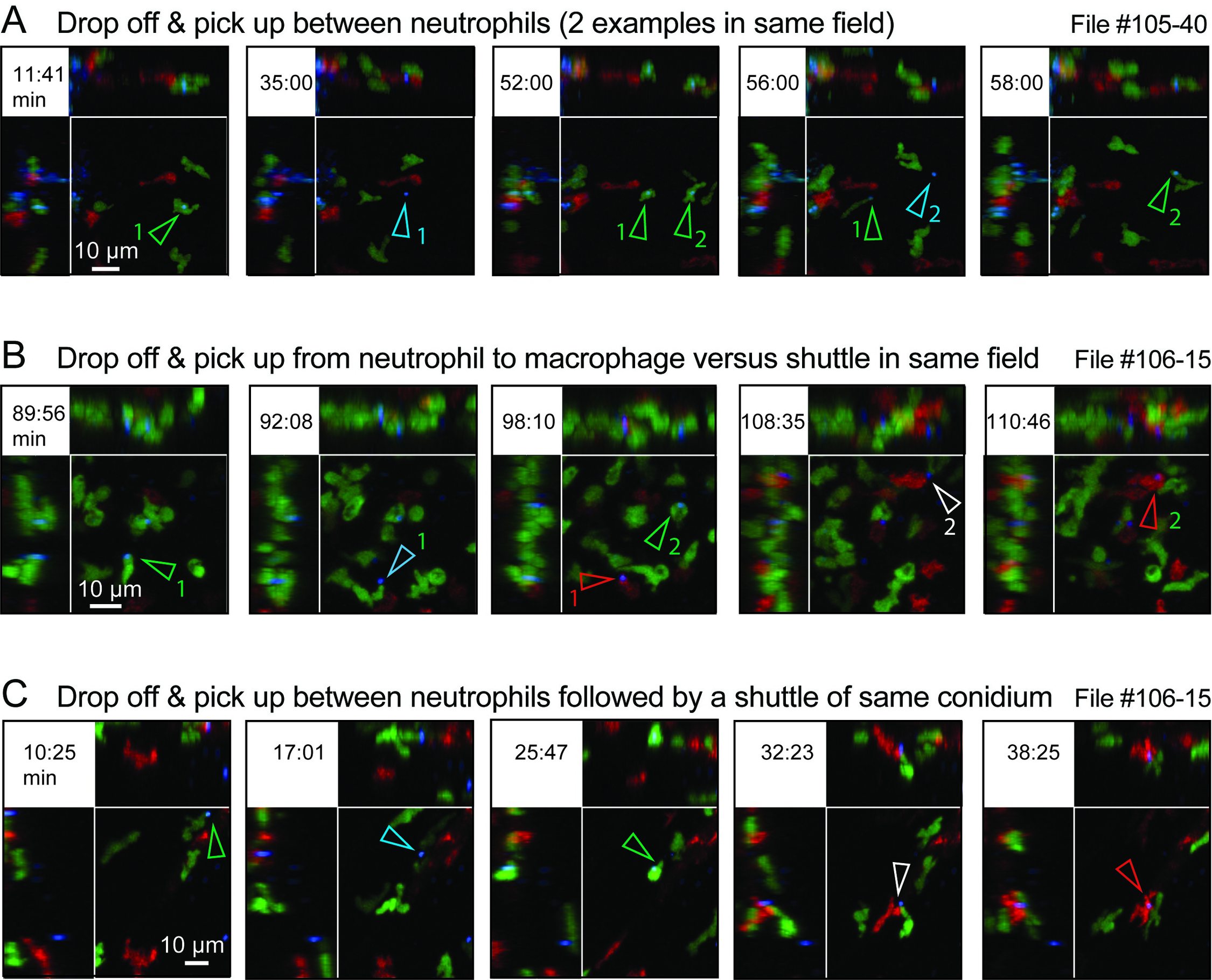

Supplement: S5 Fig — These examples are from experiments using Δgel1Δgel7Δcwh41 A. fumigatus conidia. (A) Neutrophil-to-neutrophil transfer involving conidial drop-off and departure by the donor neutrophil and reuptake of the deposited conidium by a second neutrophil, two examples in the same field of view. (B) Neutrophil-to-macrophage transfer involving conidial drop-off and departure by the donor neutrophil and reuptake of the deposited conidium by a macrophage, coincidentally occurring in proximity to a bona fide shuttle occurring slightly later. (C) Neutrophil-to-neutrophil transfer via drop-off as in (A), followed by subsequent shuttling of the same conidium from the second neutrophil to a macrophage. Arrowheads indicate the conidium of interest (numbered 1,2 where necessary) within the neutrophil (green), during the extracellular drop-off period (blue), at the time of shuttling (white), and within the macrophage (red). Scales as shown. Stills from S7A–S7C Movie. cwh41, A. fumigatus α-glucosidase 1; gel, A. fumigatus β-1,3-glucanosyltransferase. (TIF) [file pbio.3000113.s005.tif]
