# Supplementary material for: β-glucan–dependent shuttling of conidia from neutrophils to macrophages occurs during fungal infection establishment
Source: PLoS Biol. 2019 Sep 4;17(9):e3000113. doi: 10.1371/journal.pbio.3000113 (PMC6746390; doi:10.1371/journal.pbio.3000113)
Supplement: S1 Table — (DOCX) [file pbio.3000113.s013.docx]

**S1 Table. List of shuttles.**

| **Type of particle** | **#** | **Movie ID** | **comment** | **Reporter line** | **Figure** | **Movie** |
| --- | --- | --- | --- | --- | --- | --- |
| *Tm (live)* | 1 | 7 | Standard shuttle | 1 | 1A-D, 4Bi-ii | S1A |
|  | 2 | 8-1 | Standard shuttle | 1 | - | - |
|  | 3 | 8-2 | Standard shuttle | 1 | - | - |
|  | 4 | 18 | Standard shuttle | 1 | - | - |
|  | 5 | 25 | Elongated morphology of donor neutrophil | 2 | - | S1D |
|  | 6 | 27 | 2 spores shuttled asynchronously | 2 | - | - |
|  | 7 | 31 | Standard shuttle | 2 | - | - |
|  | 8 | 43 | Elongated morphology of recipient macrophage | 2 | 1E-F | S1B |
|  | 9 | 46 | Standard shuttle | 2 | 2B | S1E |
|  | 10 | 48 | Polarised morphology of donor neutrophil + cytoplasm transfer | 2 | 2A | S1C |
|  | 11 | 57 | 2 spores shuttled asynchronously | 2 | 2C | S1F |
|  | 12 | 59-3 | Standard shuttle | 2 | - | - |
|  | 13 | 59-4 | Standard shuttle | 2 | - | - |
| *Tm (dead)* | 14 | FE-1 | Three dead  *T. marneffei* shuttles in membrane labeled *CaaX* line | 3 | 3A-D | S3A-C |
|  | 15 | FE-2 | Dead *T. marneffei* shuttle in membrane labeled *CaaX* line | 3 | - | S3D |
| *Af (live)* | 16 | 60-1 | 2 spores shuttled together | 2 | - | S2F |
|  | 17 | 61-3 | Multiple contacts between cells | 2 | 2D | S2A |
|  | 18 | 63-1 | Standard shuttle | 2 | - | S2B |
|  | 19 | 63-4 x2 | Two independent shuttles in the same field | 2 | 2F | S2E |
|  | 20 | 66-48 | Standard shuttle (Alexa fluor 405 stained conidia) | 2 | 2E | S2C |
|  | 21 | 66-50 | Elongated morphology of donor neutrophil | 2 | - | S2D |
| *Af (dead)* | 22 | 68-5 | Standard shuttle | 2 | - | - |
|  | 23 | 70-1 | Standard shuttle | 2 | - | - |
| *Zymosan* | 24 | 88-3 | Shuttle at t=299 min | 2 | 6C | S5A |
|  | 25 | 111..19 | Shuttle at t=69 min | 2 | - | - |
|  | 26 | 111..44 | Shuttle at t=90 min | 2 | - | - |
| *Beads*  *+β-glucan* | 27 | 90..26 | Shuttle at t=30 min | 2 | - | - |
|  | 28 | 90..20 | Shuttle at t=169 min | 2 | 6D | S5C |
|  | 29 | 91..37 | Shuttle at t=50 min | 2 | - | - |
|  | 30 | 91..20 | Shuttle at t=57 min | 2 | - | - |
|  | 31 | 93..38 | Shuttle at t=16 min | 2 | - | - |
|  | 32 | 93.. 58 | Shuttle at t=190 min | 2 | - | - |
|  | 33 | 93..11 | Shuttle at t=46 min | 2 | - | - |
|  | 34 | 93.. 24 | Shuttle at t=238 min | 2 | - | - |
|  | 35 | 94.33 | Shuttle at t=64 min | 2 | - | - |
|  | 36 | 94..46 | Shuttle at t=57 min | 2 | - | - |
| *Beads*  *+β-glucan*  *+glucanase* | 37 | 109..27 | Shuttle at t=135 min | 2 | - | - |
|  | 38 | 109..52 | Shuttle at t=82 min | 2 | - | - |
|  | 39 | 109..50 | Shuttle at t=78 min | 2 | - | - |
| *Mutant Af* | 40 | 102..36 | Shuttle at t=34 min | 2 | - | - |
|  | 41 | 104..06 | Shuttle at t=67 min | 2 | - | - |
|  | 42 | 106..04 | Shuttle at t=111 min | 2 | - | - |
|  | 43 | 106..15 | Shuttle at t=33 min | 2 | - | - |
| *Zymosan* | 44 | Shuttled by mammalian cells (*in vitro)* | | | 7A | S6A |
|  | 45 | Shuttled by mammalian cells (*in vitro)* | | | 7B | S6B |
| *Zymosan-pHrodo*  *WT→WT* | 46 | “Exp121618” n=66 shuttles by murine cells (*in vitro)* | | | 8A,B,C | S6CDE |
|  | 47 | “Exp121718” n=98 shuttles by murine cells (*in vitro)* | | | - | - |
| *Zymosan-pHrodo*  *(testing Dectin-1^-/-^ macrophages)* | 48 | “Exp052819” n=3 WT*→* WT control shuttles (*in vitro)* | | | - | - |
|  | 49 | “Exp052819” n=1 WT*→* Dectin-1^-/-^ shuttle (*in vitro)* | | | 8F | S6F |
|  | 50 | “Exp071519” n=52 WT*→* WT control shuttles (*in vitro)* | | | Table 1 | - |
|  | 51 | “Exp071519” n=14 WT*→* Dectin-1^-/-^ shuttles (*in vitro)* | | | Table 1 | - |
|  | 52 | “Exp071619” n=37 WT*→* WT control shuttles (*in vitro)* | | | Table 1 | - |
|  | 53 | “Exp0571619” n=30 WT*→* Dectin-1^-/-^ shuttles (*in vitro)* | | | Table 1 | - |

Abbreviations:

*Tm, Talaromyces marneffei; Af, Aspergillus fumigatus; WT, wildtype.*

Reporter lines:

1. *Tg(mpeg1:mCherry/mpx:EGFP)*
2. *Tg(mpeg1*:*Gal4FF/UAS-E1b*:*Eco*.*nfsB-mCherry/mpx*:*EGFP)*
3. *Tg(mpeg1:mCherry-CaaX/mpx:EGFP-CaaX)*
