# Supplementary material for: β-glucan–dependent shuttling of conidia from neutrophils to macrophages occurs during fungal infection establishment
Source: PLoS Biol. 2019 Sep 4;17(9):e3000113. doi: 10.1371/journal.pbio.3000113 (PMC6746390; doi:10.1371/journal.pbio.3000113)
Supplement: S2 Table — (DOCX) [file pbio.3000113.s014.docx]

**S2 TABLE. Shuttling incidence for *in vitro* assays with zymosan-pHrodo and wildtype murine neutrophils and macrophages.**

| **Experiment** | **Phagocyte densities (per mm^2^)** | | | **Shuttle incidence** | | |
| --- | --- | --- | --- | --- | --- | --- |
|  | **Phagocyte type** | **t = 15 min*** | **t = time of shuttle ¶** | **n** | **per mm^2^** | **per 100 loaded neutrophils**  **at t = 15 min** |
| 1  Bone marrow neutrophils | Neutrophil - total | 506.9 ± 182.5 | 613.0 ± 217.1 | 66 | 11.2 | 4.8 |
|  | Neutrophil - loaded | 234.3 ± 93.8 | 282.4 ± 102.0 |  |  |  |
|  | Macrophage - total | 176.3 ± 75.1 | 200.0 ± 68.6 |  |  |  |
|  | Macrophage - loaded | 0.8 ± 3.2 | 53.0 ± 40.0 |  |  |  |
| 2  Peripheral blood neutrophils | Neutrophil - total | 1769.8 ± 373.9 | 1833.4 ± 338.8 | 98 | 16.7 | 20.0 |
|  | Neutrophil - loaded | 83.2 ± 39.2 | 112.6 ± 39.2 |  |  |  |
|  | Macrophage - total | 351.0 ± 197.5 | 424.5 ± 215.5 |  |  |  |
|  | Macrophage - loaded | 66.1 ± 44.9 | 214.7 ± 154.3 |  |  |  |

The total imaged area was divided into 48 subregions for scoring (3 randomly-selected imaged fields, each divided into 16 subregions).

* mean ± SD for 48 scored subregions encompassing the total imaged field

¶ mean ± SD for those individual subregions that ultimately contained a shuttle, rescored at the time of the shuttle

Datasets provided in S2 Data.
